# Rosa26-LSL-dCas9-VPR: a versatile mouse model for tissue specific and simultaneous activation of multiple genes for drug discovery

**DOI:** 10.1038/s41598-022-23127-7

**Published:** 2022-11-10

**Authors:** Dalia Pakalniškytė, Tanja Schönberger, Benjamin Strobel, Birgit Stierstorfer, Thorsten Lamla, Michael Schuler, Martin Lenter

**Affiliations:** 1 Boehringer Ingelheim Pharma GmbH & Co. KG, Drug Discovery Sciences, 88400 Biberach an der Riß, Germany; 2 Boehringer Ingelheim Pharma GmbH & Co. KG, Nonclinical Drug Safety Germany, 88400 Biberach an der Riß, Germany; 3 Boehringer Ingelheim Pharma GmbH & Co. KG, Discovery Research Coordination, 88400 Biberach an der Riß, Germany

**Keywords:** Transgenic organisms, Gene expression

## Abstract

Transgenic animals with increased or abrogated target gene expression are powerful tools for drug discovery research. Here, we developed a CRISPR-based Rosa26-LSL-dCas9-VPR mouse model for targeted induction of endogenous gene expression using different Adeno-associated virus (AAV) capsid variants for tissue-specific gRNAs delivery. To show applicability of the model, we targeted low-density lipoprotein receptor (LDLR) and proprotein convertase subtilisin/kexin type 9 (PCSK9), either individually or together. We induced up to ninefold higher expression of hepatocellular proteins. In consequence of LDLR upregulation, plasma LDL levels almost abolished, whereas upregulation of PCSK9 led to increased plasma LDL and cholesterol levels. Strikingly, simultaneous upregulation of both LDLR and PCSK9 resulted in almost unaltered LDL levels. Additionally, we used our model to achieve expression of all α_1_-Antitrypsin (AAT) gene paralogues simultaneously. These results show the potential of our model as a versatile tool for optimized targeted gene expression, alone or in combination.

## Introduction

Genetically modified mice, as in vivo models to study human disease mechanisms, have a long history that started by the end of the twentieth century^1^. Since that time, considerable technical advances and new technologies have revolutionized our ability to manipulate the mouse genome and enhance the potential of these models to support preclinical drug discovery. In particular, the application of endonucleases has greatly enhanced the feasibility for researchers to manipulate the mouse genome as desired^2,3^. Amongst them, the most widely used is the RNA-guided endonuclease CRISPR associated protein 9 (Cas9) system, which induces DNA double strand breaks with high specificity^4–6^. The specificity is provided by a short 20 base pair spacer sequence of a guide RNA (gRNA) that recognizes the target DNA region of interest and directs the nuclease for editing. Over the last few years, the CRISPR-Cas9 system was adopted, and several Cas9 variants have been generated that lack endonuclease activity, while retaining specificity for target DNA, for applications beyond classical endonuclease activity^5,7,8^. One of them is the CRISPR activation (CRISPRa) system, where endonuclease dead *Cas9* (*dCas9*) is fused with four tandem copies of Herpes Simplex Viral Protein 16 (VP64), human NF-kB p65 activation domain (p65), and Epstein-Barr Virus-derived R transactivator (Rta) domains to obtain a programmable transcription factor, termed VPR^8–10^. This hybrid dCas9-VPR was demonstrated to have a highly efficient potential for activating gene transcription of almost any gene of interest in various species and cell types and led to the development of corresponding dCas9-VPR expressing mouse models^11–14^. Here, the targeted transcriptional regulation of genes is obtained by delivering appropriate gRNAs complementary to the promoter region of the gene of interest.

Amongst various delivery methods, recombinant AAV (rAAV) is one of the most investigated and preferred tools, due to its relative safety, low immunogenicity, and ability to transduce a broad range of cells^15^. In addition, rAAV is replication defective, does not integrate into the host genome, and persists in transduced cells in an episomal fashion, thereby providing long-term transgene expression^15–17^. Moreover, due to the broad range of natural and capsid-engineered rAAV variants that differ in their transduction efficiency and tissue tropism, transgene delivery to specific cell or tissue types can be achieved^18,19^. One of the most efficient AAV serotypes for liver transduction in mice is AAV8, which was shown to transduce up to 90–95% of hepatocytes subsequent to intraportal vein or intravenous injection^20^.

The liver is one of the most important organs in the body, which is directly or indirectly involved in many essential physiological processes, where reduction or loss of liver function can be life threatening^25–33^. Hence, liver associated enzymes, circulating proteins and cell receptors are popular targets in the focus of ongoing drug discovery approaches^21–28^. In this context, hepatocyte expressed LDLR plays an important role in plasma cholesterol homeostasis, where dysregulation leads to a higher risk for the development of cardiovascular diseases^29^ LDLR is located at the cell surface of hepatocytes, where it interacts with plasma derived LDL. After binding, the LDLR-LDL complex is internalized and transported into endosomes^29^. Once LDL has been released, LDLR can recirculate to the cell surface or is degraded in the lysosomes. The degradation rate of LDLR can be regulated by modifying another enzyme (PCSK9), which is mainly liver expressed^30–32^. PCSK9 interacts with LDLR on the cell surface and targets LDLR to lysosomes for its degradation^29^. It has been shown that high levels of circulating PCSK9 increase concentrations of plasma LDL, increasing the risk of atherosclerosis development^33–35^.

Another important protein expressed by hepatocytes is AAT, which is encoded by the *SERPINA1* gene. Liver secreted AAT circulates in the blood and its main function is to control activity of various serine proteinases. The primary target of AAT is neutrophil elastase^36,37^. The number of *SERPINA1* genes vary among different mammalian species. Primates, including humans, and some mouse strains contain only a single gene copy, while other mouse lines contain multiple paralogues originating from the same ancestral gene. For instance, C57Bl/6 J mice contain five *Serpina1* paralogues, namely *Serpina1a*, *Serpina1b*, *Serpina1c*, *Serpina1d* and *Serpina1e*^36,38–40^.

Here, we describe the development and use of a new Cre recombinase-dependent dCas9-VPR mouse model, with the potential of long-lasting transcriptional activation in vivo. This mouse model is applicable for gene induction by gRNAs to target different genes of interest, individually or in combination. Particularly, by using liver tropic AAV8 and specific gRNAs, we demonstrate tissue specific upregulation of LDLR, PCSK9 and AAT in hepatocytes. For AAT, all five *Serpina1* gene variants could be simultaneously upregulated in our mouse model using a mix of five AAV8 preparations, each containing specific gRNAs against one of the five *Serpina1* gene variants. Conditions of hyper- or hypocholesterolemia were successfully induced in these mice by activating the expression of either hepatic PCSK9 and/or LDLR. Taken together, these studies demonstrate the potential of the new Rosa26-LSL-dCas9-VPR mouse model for targeted transcriptional gene activation, thereby enabling rapid characterization and validation of gene function in basic biological research or drug discovery.

## Results

### Generation of a Cre-dependent dCas9-VPR knock-in transgenic mouse line

**Figure 1:**
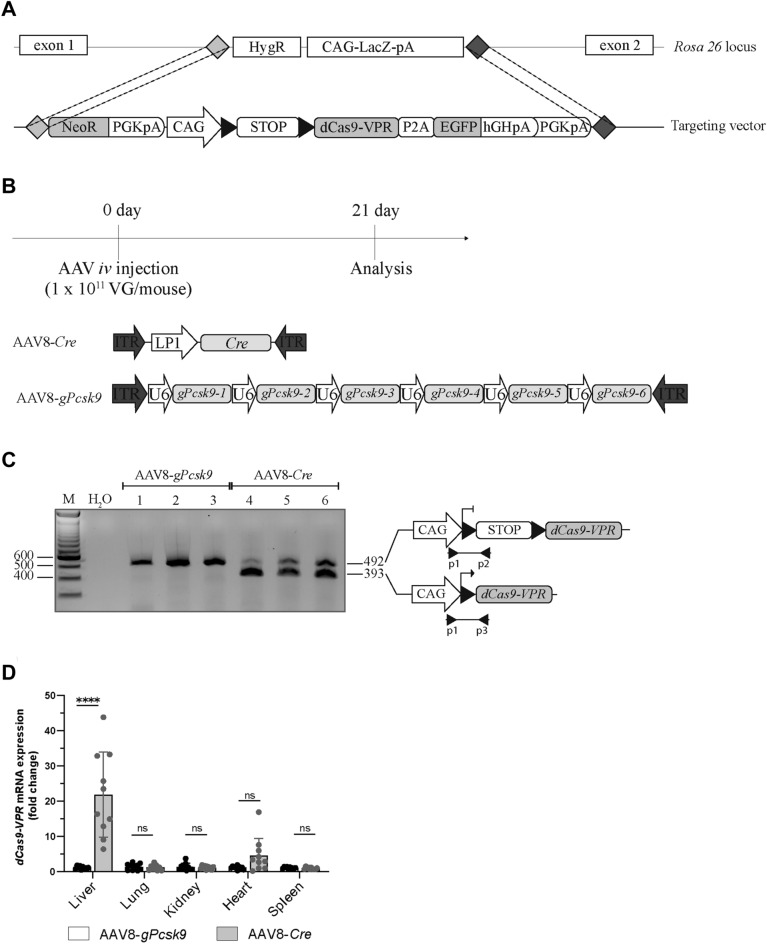
Generation and characterization of Cre-dependent dCas9-VPR-expressing mice. (**A**) Scheme of the Cre-dependent dCas9-VPR Rosa26 targeting vector. (**B**) Outline of the in vivo experiment. Rosa26-LSL-dCas9-VPR mice were *iv* injected with AAV8 containing *LP1-Cre* gene (AAV8-*Cre*) or 6 different gRNAs against *Pcsk9* (AAV8-*gPcsk9*), named *gPcsk9-1 to 6*, at the amount of 1 × 10^11^ VG/mouse. 21 days later mice were sacrificed, and tissues were collected for analysis. (**C**) PCR representation showing LSL cassette recombination in liver tissues isolated from Rosa26-LSL-dCas9-VPR mice transduced with AAV8-*Cre* or AAV8-*gPCSK9* alone. A representative agarose gel electrophoresis image is shown. Lane 1–3 contains amplicons obtained from tissue samples of three different mice treated with AAV8-*gPcsk9* and lanes 4–6 from tissue samples of three different mice treated with AAV8-*Cre*. The expected size of PCR products, marker (M) and NTC (H2O) are indicated. (**D**) dCas9-VPR RNA expression in tissues dissected from AAV8-*Cre* or AAV8-*gPCSK9* injected Rosa26-LSL-dCas9-VPR mice. dCas9-VPR expression can only be seen in AAV8-*Cre* treated Rosa26-LSL-dCas9-VPR mice liver samples. Mean values are shown as a relative quantification, with corresponding expression level of AAV-*Cre* treated control group as a reference (n = 10).

We generated the Cre-dependent CRISPR activation mouse line, termed Rosa26-LSL-dCas9-VPR, using the recombinase mediated cassette exchange technology to integrate the dCas9-VPR gene containing cassette into the *ROSA26* locus (Fig. 1A). The cloned targeting vector contained a NeoR cassette, the human CAG promoter, and a translation interrupting LSL cassette linked with the *dCas9-VPR* gene fused to a self-cleaving P2A sequence and *Egfp* gene (Fig. 1A).

### Characterization of Cre-dependent and tissue-specific dCas9-VPR expression

To investigate the effectiveness of Cre recombinase-mediated LSL cassette excision, and consequently dCas9-VPR expression activation, in a tissue specific manner, Rosa26-LSL-dCas9-VPR mice were either injected with a preferentially liver transducing AAV8 containing the *Cre* gene under the control of a liver specific *LP1* promoter (AAV8-*Cre*) or AAV8 carrying U6 promoter driven guide RNAs targeting Pcsk9 (*gPcsk9)* (AAV8-*gPcsk9*), where each gRNA is driven by separate U6 promoter (Fig. 1B). Recombination (i.e., excision of the LSL cassette) was only observed in liver tissue of AAV8-*Cre* administered Rosa26-LSL-dCas9-VPR mice, but not in solely AAV8-*gPcsk9* transduced mice (Fig. 1C). Moreover, in AAV8-*Cre* treated mice, dCas9-VPR expression was restricted to liver, with absent or only minor but not statistically significant increases in all other investigated tissues, including heart, lung, kidney, and spleen (Fig. 1D). These results show that Rosa26-LSL-dCas9-VPR mice in combination with AAV8 and LP1 promoter driven Cre expression allow efficient and liver specific induction of dCas9-VPR expression.

### Upregulation of *Serpina1* paralogues in the liver by CRISPRa

**Figure 2:**
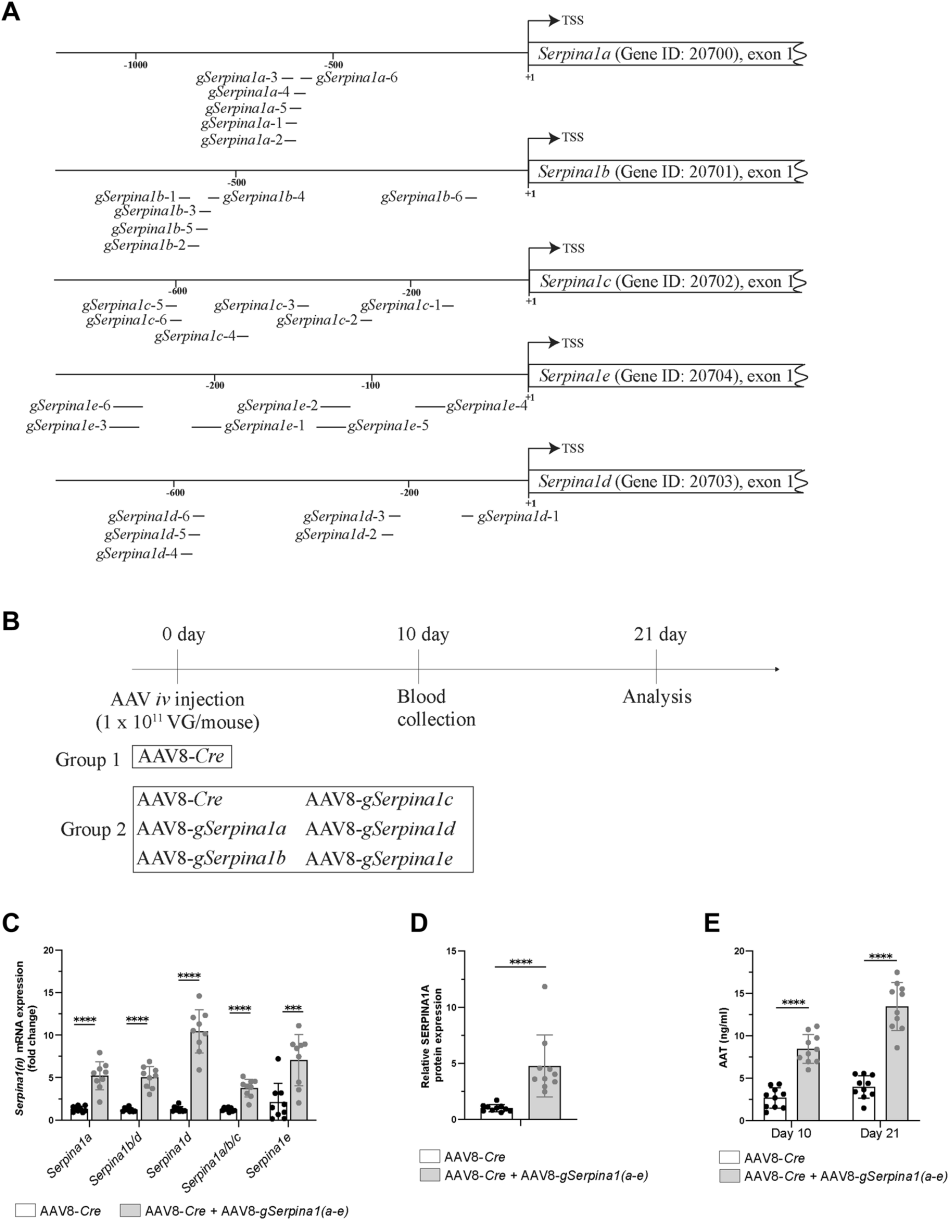
Multiple gRNAs facilitate the transcription of the endogenous mouse *Serpina1(a-e)* genes in vivo. (**A**) Relative position of the 6 individual gRNAs (black bars) per Serpina1 (a-e) paralogue, aiming to upregulate transcription, are shown. The transcription start site (TSS) in the promoter region is shown as a black arrow. The numbers indicate the base pair distance to the TSS. The position where individual gRNAs bind to the genome is displayed relative to the TSS of the respective *Serpina1* transcript (Serpina1a-203 (ENSMUST00000124717.2), Serpina1b-201 (ENSMUST00000095450.11), Serpina1c-201 (ENSMUST00000074051.6), Serpina1e-202 (ENSMUST00000122229.8), Serpina1d-201 (ENSMUST00000078869.6)). (**B**) Outline of the in vivo experiment. Rosa26-LSL-dCas9-VPR mice were *iv* injected with AAV8 containing *LP1-Cre* (AAV8-*Cre*) alone or in parallel with a set of 5 different AAV8s, carrying gRNAs against 5 Serpina1 paralogues, at the amount of 1 × 10^11^ VG/mouse. 21 days later mice were sacrificed, and tissues were collected for analysis. (**C**,D) *Serpina1(a-e)* mRNA and protein expression in liver tissue dissected from Rosa26-LSL-dCas9-VPR mice, treated with only AAV8-*Cre* or with a set of six different viruses, AAV8-*Cre*, AAV8-*gSerpina1a*, AAV8-*gSerpina1b*, AAV8-*gSerpina1c*, AAV8-*gSerpina1d* and AAV8-*gSerpina1e*. Mean values are shown as a relative quantification, with corresponding expression level of AAV-*Cre* treated control group as a reference (n = 9–10). (**E**) AAT protein levels in mouse plasma from Rosa26-LSL-dCas9-VPR mice. Means and standard deviations are shown, n = 10.

To determine whether the Rosa26 knock-in construct provided functional levels of dCas9-VPR expression, we next investigated parallel transduction of CRISPRa mice with AAV8-*Cre* and a set of five AAV8s containing *gRNAs* targeting the five *Serpina1* paralogues a-e (AAV8-*gSerpina1a*, AAV8-*gSerpina1b*, AAV8-*gSerpina1c*, AAV8-*gSerpina1d*, AAV8-*gSerpina1e)* (Supplementary Fig. S1). Each paralogue is targeted in parallel by 6 different gRNAs, where each gRNA is driven by an individual U6 promoter (Fig. 2A). We injected two groups of animals and collected blood samples 10- and 21-days post transduction. While the animals of the first group received only AAV8-*Cre*, the second group received a combination of AAV8-*Cre* and all five AAV8-*gSerpina1*, each targeting one of the five *Serpina1* gene variants a-e (AAV8-*gSerpina1(a1-6-e1-6)*), with the aim to upregulate all 5 liver *Serpina1* paralogues simultaneously in each animal (Fig. 2B). 21 days post transduction, Rosa26-LSL-dCas9-VPR mice were investigated for AAT expression in the liver and blood. mRNA analysis of all *Serpina1* variants at day 21 showed an increased expression in the liver of mice that received both *Cre* and *Serpina1 gRNAs*, demonstrating that dCas9-VPR expression was successfully induced and capable to form a ribonucleoprotein complex (RNP) with the provided gRNAs to facilitate on-target gene over-expression (Fig. 2C). Furthermore, we performed protein analysis of liver samples and found a fivefold overexpression of the SERPINA1A paralogue as compared to animals receiving only AAV8-*Cre* (Fig. 2D). To confirm this observation, we additionally quantified AAT levels in plasma (Fig. 2E). In line with the results described above, injection of mice with AAV8-*Cre* and AAV8-*gSerpina(n)* led to significantly increased AAT plasma levels detected on day 10 post-transduction, which further increased on day 21 post injection, while control levels remained almost unchanged (Fig. 2E). Taken together, these data demonstrate the successful transcriptional induction of all five *Serpina1* paralogues, thereby providing evidence for the use of the Rosa26-LSL-dCas9-VPR mice in combination with AAV8 encoded gRNAs for efficient upregulation of *Serpina1* transcription resulting in increased protein levels in liver and plasma.

### Individual and simultaneous upregulation of LDLR and PCSK9

**Figure 3:**
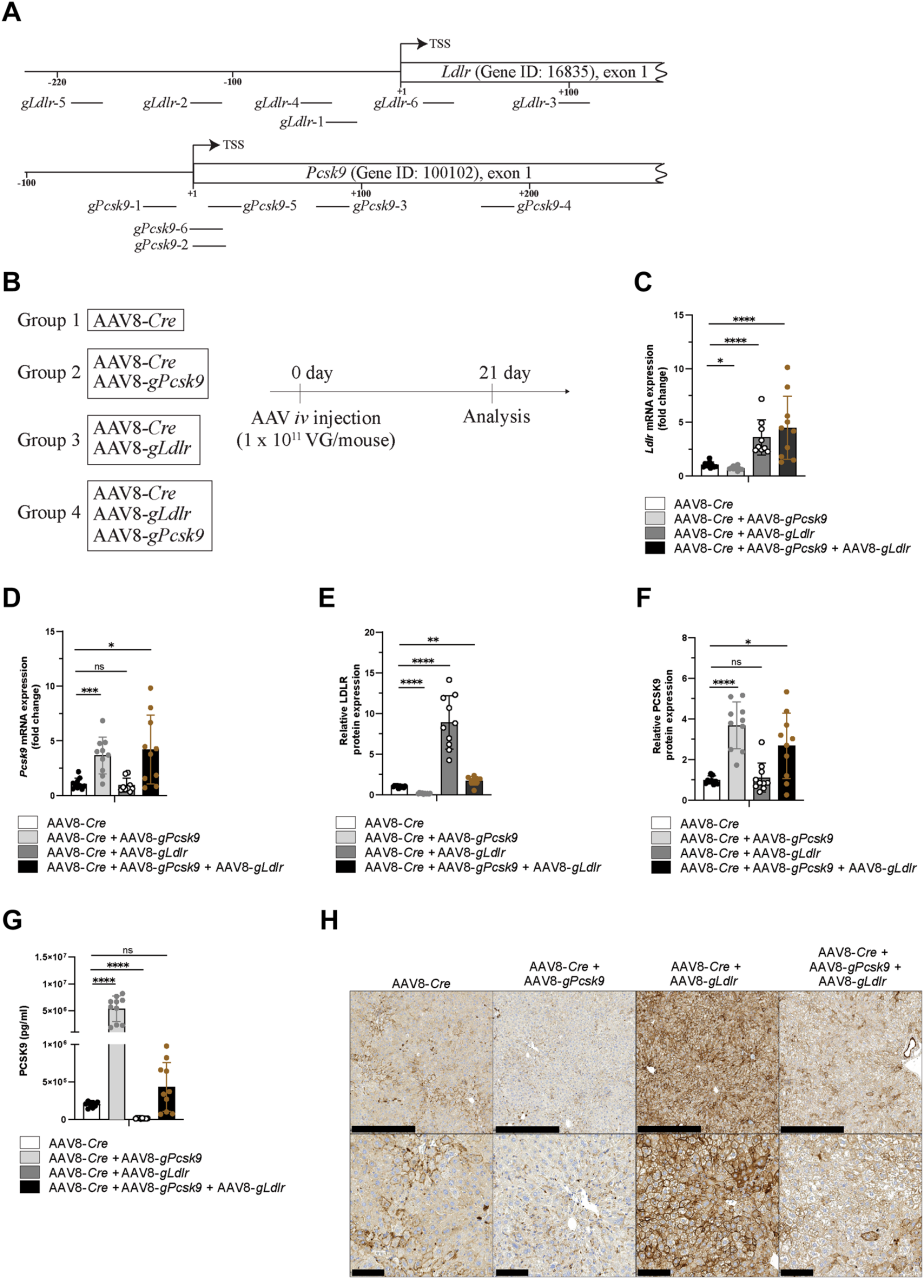
Parallel *Ldlr* and *Pcsk9* overexpression in vivo using AAV8-transduced CRISPRa mice. (**A**) Relative localization of the 6 individual gRNAs (black bars) targeting mouse *Pcsk9* and *Ldlr* genes are depicted. The transcription start site (TSS) in the promoter regions are shown as black arrows. The numbers indicate the distance in base pair relative to the TSS. The position where individual gRNAs bind to the genome is displayed relative to TSS of the respective *Ldlr* and *Pcsk9* transcripts (Ldlr-201 (ENSMUST00000034713.9), Pcsk9-201 (ENSMUST00000049507.6)). (**B**) Outline of the in vivo experiment. Rosa26-LSL-dCas9-VPR mice were *iv* injected with AAV8 containing *LP1-Cre* (AAV8-*Cre*) alone or in combination with AAV8-*gLdlr*, AAV8-*gPcsk9* or a mixture of both, at the amount of 1 × 10^11^ VG/mouse. 21 days later mice were sacrificed, and tissues were collected for analysis. (**C**,**D**) *Ldlr* and *Pcsk9* mRNA expression in liver tissue isolated from Rosa26-LSL-dCas9-VPR mice treated with AAV8-*Cre* alone, or in combination with AAV8-*gLdlr*, AAV8-*gPcsk9* or a mixture of both. Mean values are shown as a relative quantification, with corresponding expression level of AAV-*Cre* treated control group as a reference (n = 9–10). (**E**) *Ldlr* protein expression in liver tissue isolated from Rosa26-LSL-dCas9-VPR mice treated with AAV8-*Cre* alone, or in combination with AAV8-*gLdlr*, AAV8-*gPcsk9* or a mixture of both. Mean values are shown as a relative quantification, with corresponding expression level of AAV-*Cre* treated control group as a reference (n = 9–10). (**F**) *Pcsk9* protein expression in liver tissue isolated from Rosa26-LSL-dCas9-VPR mice were treated with AAV8-*Cre* alone, or in combination with AAV8-*gLdlr*, AAV8-*gPcsk9* or a mixture of both. Mean values are shown as a relative quantification, with corresponding expression level of AAV-*Cre* treated control group as a reference (n = 10). (**G**) PCSK9 protein levels detected in mouse plasma from Rosa26-LSL-dCas9-VPR mice. Means and standard deviations are shown, n = 10. (**H**) Representative images of anti-LDL receptor stained liver sections showing differential LDLR expression in Rosa26-LSL-dCas9-VPR mice injected with AAV-*Cre* alone, together with AAV-*gLdlr* or AAV8-*gPcsk9*, or simultaneous treatment. Upper panel scale bar, 500 µm. Lower panel, magnified, scale bar, 100 µm.

A key advantage of CRISPR-based models is their potential for targeting of two or more genes at the same time by combining different gRNAs. To study the possibility of multiple gene upregulation in our Cre-dependent dCas9-VPR mice, we selected the well characterized PCSK9-LDLR-LDL regulatory loop for the next experiment. PCSK9 plays an important role in cholesterol homeostasis by forming a complex with LDLR on the cell surface, thereby inducing LDLR’s internalization and subsequent lysosomal degradation. To induce PCSK9 and LDLR expression, we selected a set of 6 different gRNAs, each targeting either *Ldlr* or *Pcsk9* (Fig. 3A). To study the function of both genes on cholesterol homeostasis, we analyzed liver and blood 21 days post AAV injection. Animals of the first group were injected with AAV8-*Cre* alone, as a control, whereas the second and third groups additionally received either AAV8-*gPcsk9* or AAV8-*gLdlr,* respectively (Supplementary Fig. S1). The fourth group was injected with a combination of three different viruses: AAV8-*Cre*, AAV8-*gLdlr* and AAV8-*gPcsk9* (Fig. 3B). As expected, treatment with AAV8-*Cre* either in combination with AAV8-*gLdlr* or AAV8-*gPcsk9* led to a significant, approximately threefold transcriptional upregulation of either *Ldlr* or *Pcsk9*, compared to the control group (Fig. 3C,D). In group four (AAV8-*Cre* + AAV8-*gLdlr* + AAV8-*gPcsk9*), the transcriptional upregulation of both *Ldlr* and *Pcsk9* was comparable to the individual induction of *Pcsk9* expression in group two (AAV8-*Cre* + AAV8-*gPcsk9*) or *Ldlr* in group three (AAV8-*Cre* + AAV8-*gLdlr*) (Fig. 3C,D). The increase in mRNA translated into an even more pronounced upregulation on protein levels, with a ninefold or fourfold overexpression for LDLR or PCSK9, respectively (Fig. 3E,F). Specificity of PCSK9 detection via Wes™ was confirmed using recombinant PCSK9 protein. Virtual blot-like images are shown in Supplementary Fig. S2. Upregulation of PCSK9 reduced LDLR protein in liver tissue by tenfold (Fig. 3E, AAV8-*Cre* + AAV8-*gPcsk9*), whereas upregulation of LDLR protein did not affect the PCSK9 protein amount in liver tissue (Fig. 3F, AAV8-*Cre* + AAV8-*gLdlr*), but reduced circulating PCSK9 levels in plasma by 13-fold (Fig. 3G, AAV8-*Cre* + AAV8-*gLdlr*). Simultaneous upregulation of PCSK9 and LDLR proteins led to an almost twofold increase in LDLR protein amount in liver (Fig. 3E, AAV8-*Cre* + AAV8-*gLdlr* + AAV8-*gPcsk9*), whereas the detected PCSK9 amounts were increased almost threefold compared to the control group (Fig. 3F, AAV8-*Cre* + AAV8-*gLdlr* + AAV8-*gPcsk9*). However, despite these higher PCSK9 protein amounts observed in the liver tissue lysates, no significant PCSK9 increase was detected in the corresponding plasma samples (Fig. 3G, AAV8-*Cre* + AAV8-*gLdlr* + AAV8-*gPcsk9*). To confirm the increase of LDLR on the surface of the hepatocytes from the dCas9-VPR expressing mice, we additionally performed immunohistochemistry analyses on liver sections stained with an anti-LDLR antibody. The antibody dilution was titrated to see a moderate staining in the control group showing faint cytoplasmic staining and, in a fraction of hepatocytes, distinct membrane staining. As expected, upregulation of PCSK9 in the experimental group two led to decreased LDLR staining compared to the control group (Fig. 3H, AAV8-*Cre* + AAV8-*gPcsk9*), whereas upregulation of the LDL-receptor resulted in a strong increase in membrane staining and, to a lesser extent, in cytoplasmic staining (AAV8-*Cre* + AAV8-*gLdlr*). After simultaneous upregulation of both PCSK9 and LDLR, the staining for LDLR levels is comparable to the control group (Fig. 3H, AAV8-*Cre* + AAV8-*gLdlr* + AAV8-*gPcsk9*).

### Regulation of plasma cholesterol by modulating hepatic LDLR expression

**Figure 4:**
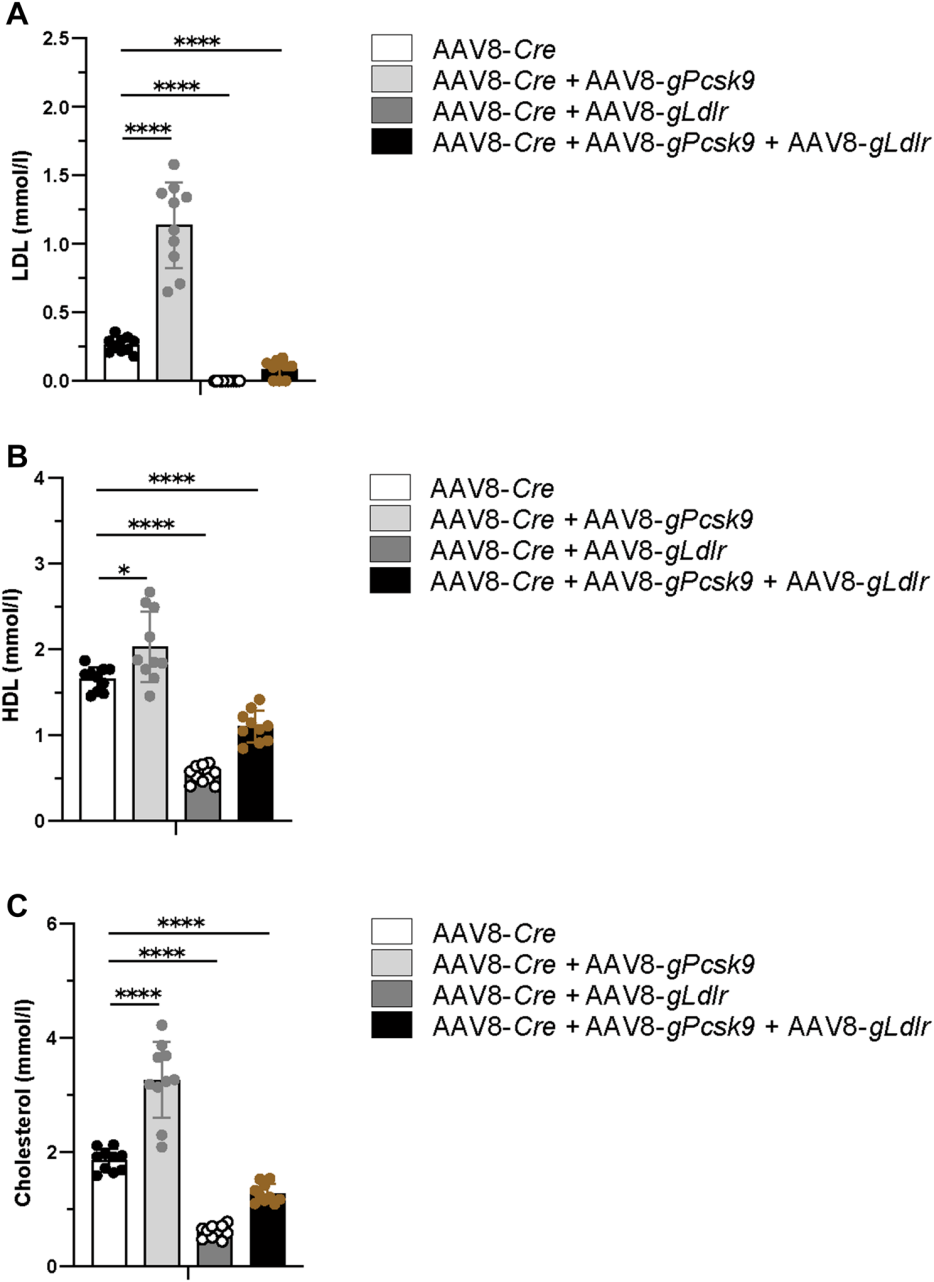
Plasma LDL, HDL and cholesterol concentrations in Rosa26-LSL-dCas9-VPR mice after AAV8-*gLdlr/gPcsk9* mediated upregulation of hepatic LDLR and/or PCSK9. (**A**,**B**) Concentrations of LDL and HDL in plasma from Rosa26-LSL-dCas9-VPR mice. Mean values of concentrations and standard deviations are shown, n = 10. (**C**) Total cholesterol concentrations in plasma from Rosa26-LSL-dCas9-VPR mice. Means and standard deviations are shown, n = 10.

To evaluate the effect of hepatic LDLR overexpression, or its reduction by the enhanced PCSK9 expression, on plasma cholesterol levels, we subjected plasma of the AAV8-transduced dCas9-VPR mice to lipoprotein analysis. As expected, treatment with AAV8-*Cre* in combination with AAV8-*gLdlr* led to a significant decrease of LDL, HDL as well as cholesterol in plasma compared to the samples from the control group (Fig. 4A–C, AAV8-*Cre and* AAV8-*Cre* + AAV8-*gLdlr*). In detail, the HDL and total cholesterol levels dropped threefold, whereas plasma LDL dropped to an undetectable amount. In line with these data, AAV-mediated overexpression of PCSK9 increased LDL, HDL and total cholesterol concentrations in plasma compared to the control group samples (Fig. 4A–C, AAV8-*Cre* and AAV8-*Cre* + AAV8-*gPcsk9*). In this context, upregulation of PCSK9 resulted in fourfold higher plasma LDL levels and a twofold increase for total cholesterol when compared to the control group (AAV8-*Cre*), whereas HDL levels were almost unchanged (1.2-fold increase) (Fig. 4B). Notably, simultaneous upregulation of LDLR in parallel to PCSK9 (Fig. 4A–C, AAV8-*Cre* + AAV8-*gLdlr* + AAV8-*gPcsk9*) still resulted in a reduction of plasma LDL, HDL and cholesterol levels, but less pronounced than upon upregulation of LDLR alone. This finding is in concordance with a residual increase of LDLR levels of almost twofold compared to control, despite PCSK9 induction (Fig. 3E, AAV8-*Cre* + AAV8-*gLdlr* + AAV8-*gPcsk9* and AAV8-*Cre*).

## Discussion

In our study, we generated a novel, conditional (Cre-dependent) dCas9-VPR expressing mouse line and demonstrated its utility for tissue specific gene upregulation using AAV-mediated gRNA expression. Our mouse model offers a versatile basis for diverse research applications that require fine-tuning of targeted expression of any gene of interest using either a mixture or a single AAV with varying tissue tropism, thereby providing the opportunity to simultaneously activate multiple genes in vivo. Additionally, our model offers the advantage to restrict dCas9-VPR expression to a desired tissue by placing Cre-recombinase under a tissue-specific promoter, if needed. Alternatively, the mice could also be cross-bred with Cre driver lines to induce tissue- or cell-specific dCas9-VPR expression.

The primary goal of our study was to establish and improve a CRISPR activation model to study pathways and molecular interactions in conjunction with tailored disease models to reproduce human pathological conditions for basic research and drug development. Because of their phylogenetic relatedness and physiological similarity to humans, the use of mice as tools in biomedical research is well established^3,41–45^. Unfortunately, none of these models precisely mimic the human phenotype exactly enough, leading to variations in efficacy and toxicity of drug candidates compared to humans in the past^46–51^. Even though the genetic pathways regulating normal and physiological conditions are quite conserved, the intrinsic genetic differences sometimes complicate the direct comparison between the species^52^. One example is the generation of authentic AAT mouse-models, where the establishment of comparable disease models are hampered by the complexity of the murine *Serpina1*-genes^36,38–40^. The need for mouse models to upregulate the expression of all *Serpina1* paralogues simultaneously is of particular importance since there is evidence to suggest that overexpression of AAT is most probably involved in cancer related processes^53,54^. It has been already shown that higher AAT expression promotes invasion and metastasis as well as correlates with poor prognosis in patients with lung, colon, skin, and gastric cancer^53–57^. The mouse model presented here will therefore be highly relevant to further investigate not only these findings but can be easily adapted to similar genetic conditions.

The most important features to reproduce human diseases are the precision of etiology as well as the ability to reproduce the features of the pathological process. The value of our mouse model to induce and study the regulation of complex pathological conditions is, therefore, demonstrated by the successful modulation of cholesterol metabolism by the hepatic overexpression of two system relevant key players, namely LDLR and PCSK9, alone and in combination. In accordance with recent studies^29,30,32,34,58^, overexpression of circulating PCSK9 led to reduced LDLR, which was accompanied by increased LDL and cholesterol concentrations in plasma and vice versa. In addition to this, we also observed sex dependent differences in serum/plasma levels of alpa-1-antitrypsin (male > female), LDL (female > male) and PCSK9 in control mice in accordance with the literature^59–64^. This distinction was still visible in AAT plasma levels despite a general 1.6-fold increase after overexpression. In contrast to this, we could not detect animal sex related significant differences in liver dCas9-VPR mRNA expression. As demonstrated with these data, our model is well suited to study PCSK9 function in the liver via LDLR depletion, but moreover, also possible effects on other organs can be easier addressed, e.g., by the investigation of compensatory effects mediated via additional tissue specific LDLR upregulation.

Several CRISPRa mouse models have already been generated, which mainly differ in the choice of the transcriptional activator, chosen locus for *dCas9* gene knock-in, the target vector design, and whether dCas9 is conditionally or constitutively expressed^13,14,65–68^. We specifically decided to use dCas9 fused to VPR under the control of a CAG promoter within the *Rosa26* locus and downstream of an LSL cassette. To allow gene upregulation in any tissue of interest, we followed the strategy to use the "safe harbor" locus *Rosa26* as the preferred site for ubiquitous expression of our transgene. By doing so, we made sure to reach similar expression of dCas9-VPR across various tissues, without affecting endogenous gene expression as observed in mouse models in the past^44,69–72^. This was also done by Hunt et al., who published a CRISPRa model using the *Rosa26* locus^66^. However, in this configuration the dCas9-synergistic activation mediator (SAM) was placed exclusively under the transcriptional control of the *Rosa26* promoter, which might limit the transgene expression levels due to its moderate strength. To overcome this limitation, our (and other) CRISPRa mouse model was generated by additionally inserting a strong exogenous CAG-promoter into the *Rosa26* locus upstream of the *dCas9* transgene^14,65,73,74^. The decision to use dCas9 fused to the VPR activator was mainly fostered by a comprehensive study by Chavez et.al., where they compared different Cas9 activator systems in several human, mouse and fly cell lines^15^. Even though AAV based gRNA delivery can often be sufficient for tissue-specific target gene expression, we aimed to further increase tissue specificity in our model by conditional dCas9-VPR expression. In addition to that, transgene expression needs to be supervised to prevent unwanted side effects, as several publications pointed out a possible dCas9-VPR toxicity^14,75^. Moreover, by limiting dCas9 expression to certain tissues, also the risk of gRNA off-targeting is reduced. Narrowing down dCas9-VPR expression to defined cell types and tissues in our mouse model can be achieved by combining tissue tropism provided by AAV serotypes with tissue specific promoter driven Cre. This strategy is of special interest since the field of AAV capsid engineering is thriving and a number of additional AAV serotypes have been isolated and new capsid variants have been generated in recent years. Despite these attractive features, the CRISPRa/AAV-guide system still possesses some limitations, such as the limited availability of truly tissue or cell specific promoters for controlled Cre expression, inefficient in vivo transduction of some tissues (e.g., bone marrow, immune cells, kidney) with AAVs, and specificity of gRNAs. Nevertheless, in light of the large body of information gained from studies in mice, where different AAV serotype vectors have been shown to exhibit distinct tropism for various tissues^77^, ongoing efforts to identify novel promoter/enhancer elements for a variety of tissues, and continuous improvements of CRSIPR technology and guide design, our model holds the potential to target genes in hardly accessible tissues or cell types in the future.

Finally, we have decided to use 6 gRNA sequences to target one gene, as it was demonstrated that most efficient gene upregulation is reached when more than 3 gRNAs are used in parallel^76^. Although the selection of these gRNAs was based on a prediction algorithm that aims to select target-specific sequences, off-target effects cannot be fully excluded and therefore need to be carefully addressed in any future study using appropriate methods, e.g., ChIP-seq and prior in vitro evaluation to minimize off-target effects that might otherwise falsify data interpretation.

## Conclusions

Our Rosa26-LSL-dCas9-VPR model can be a used to study and validate pathways/molecular interactions by selected or combined overexpression of genes. It also offers the possibility for concerted overexpression of multiple gene variants in order to study their biological function jointly and/or individually. Additionally, it has the potential to generate mouse disease models by overexpression of endogenous genes based on the sustained and potentially long-lasting expression of AAV8 constructs in mouse liver^78–80^ and by combining multiple genes in order to achieve the expected disease phenotypes. The fast generation, the precise gene targeting, and the versatile combination of multiple genes makes this model highly attractive, not only for academia but also for industry, to support and accelerate drug discovery by providing detailed insights in target pathway biology and to set up new disease animal models with a better match to human pathology.

## Material and methods

### Mouse model generation animal husbandry

Rosa26-LSL-dCas9-VPR mouse line was generated by recombinase mediated cassette exchange (RMCE) technology (Taconic Bioscience). RMCE vector containing F3 site, neomycin resistance (*NeoR*) gene, PGK polyadenylation signal, cytomegalovirus (CMV) immediate enhancer/β-actin (CAG) promoter, *loxP-STOP-loxP* (LSL) cassette, *dCas9-VPR* gene, *P2A* sequence, *enhanced green fluorescent protein* (*EGFP*) gene, hGH polyadenylation signal, PGK polyadenylation signal and FRT site was cloned, and transfected into a C57BL/6NTac embryonic stem cell (ESC) line containing an RMCE docking site in the *Rosa26* locus (Taconic Bioscience). The targeted ESC clone was injected into BALB/c blastocysts. Spermatozoa from high-percentage chimeric male mice was used for in vitro fertilization of superovulated C57BL/6NTac female mice (Taconic Bioscience) to obtain a first colony of transgenic C57BL/6NTac-*Gt(ROSA)26Sor*^*tm6458 (CAG-LSL-dCas9VPR-EGFP)Tac*^ (termed Rosa26-LSL-dCas9-VPR or *Rosa26*^*LSL-dCas9-VPR/*+^) mice. Mice were housed in groups of 3–5 in individually ventilated cages at 22–25 °C, a humidity of 45–65%, and a 12 h day/night cycle with free access to water and food. Animal experiments were performed in accordance with the German Animal Welfare Act, and the guidelines of directive 2010/63/EU of the European Parliament and the Council 2010 on the protection of animals used for scientific purposes. Animal experiments performed in this study were reviewed and approved by the local authorities (Regierungspräsidium Tübingen, TVV-17-020). We hereby confirm that all methods in the study were carried out in compliance with the ARRIVE (Animal Research: Reporting of In Vivo Experiments) guidelines and regulations.

### In vivo AAV administration

AAV8 particles were resuspended in 0.9% NaCl saline (04671613, Deltamedica). 125 µl were injected into *Rosa26*^*LSL-dCas9-VPR/*+^ mice via tail vein injection at a dose of 1 × 10^11^ VG/mouse for each AAV variant. For all experiments, male and female mice at the age of 20–26 weeks were used and genders were distributed equally between the experimental groups.

### AAV expression constructs for gRNAs and Cre recombinase

gRNA and LP1-Cre sequences were cloned into separate plasmids harboring AAV2-inverted-terminal repeats (ITRs) with one ITR harboring a mutated terminal resolution site, therefore resulting in a self-complementary genome. Each gRNA expression vector contained six consecutives human U6 promoters, each followed by a gRNA coding sequence. The native *Streptococcus pyogenes*-derived gRNA scaffold structure was optimized based on previous publications^81,82^. gRNA sequences were taken from a data base, published by Horlbeck et al.^83^, and are listed in Supplementary Table S1. The Cre expression vector was composed of an LP1 promoter, *Cre* gene and an SV40 polyA sequence. Complete plasmid sequences and annotations can be found in Supplementary Table S2.

### AAV production, purification, and titration

AAVs were produced using frozen high-density HEK293 cell stocks and CELLdiscs (678116, Greiner Bio-one) as previously described^84^. Briefly, AAVs were produced by calcium phosphate transfection in a minimum of three 16-layer CELLdiscs, resulting in total yield of 2.39E+12–6.84E+12 VG, depending on the construct. Freshly thawed high-density HEK293 cell stocks were seeded with a density of 6E+07 cells per disc, followed by incubation at 37 °C for 72 h. For one disc, 1800 µg of plasmid DNA was mixed with 69 ml 300 mM calcium chloride (C7902, Sigma-Aldrich), added dropwise to 69 mL 2× HBS buffer, pH 7.0 (15450257, Thermo Fisher Scientific) and added to the cells after 2 min of incubation. Plasmid DNA contained equimolar amounts of a rep2-cap8 plasmid, pHelper (Applied Biosystems), and either Cre or one of the gRNA expression plasmids. Six hours after transfection, the medium was changed, and cells were further incubated for 72 h before harvesting. Cells were then lysed in lysis buffer, containing 1300 IU (i.e., 0.325 IU/cm^2^) salt active nuclease (70910-150, Scientifix) and HALT protease inhibitor (78439, Thermo Fisher Scientific). After spinning the cell debris, supernatants were collected and further processed by PEG-precipitation, iodixanol gradient ultracentrifugation and ultrafiltration, as described in detail before^84,85^. Droplet digital PCR for absolute quantification of viral genomes for gRNA-expressing virus was performed using an U6 promoter specific Custom TaqMan™ Gene Expression Assay (Applied Biosystems), containing U6-Fwd, U6-Rev and U6-probe (Supplementary Table S1). Absolute quantification for *Cre*-expressing AAVs was performed using primers LP1-Fwd, LP1-Rev and LP1-probe (Sigma-Aldrich) (Supplementary Table S1).

### Tissue homogenization

For protein analysis, dissected tissues were immediately snap frozen, while for RNA analysis, organs were first submerged in RNAlater™ Stabilization Solution (AM7021, ThermoFisher) for 24 h at 4 °C. Tissues were transferred into Precellys® tubes (Bertin Instruments) together with 50 µl/10 mg RLT buffer (79,216, QIAGEN) containing 1% β-mercaptoethanol (M3148, Sigma-Aldrich) for RNAlater stabilized tissues or 100 µl/10 mg RIPA buffer (R0278, Sigma-Aldrich) containing 1X HALT Protease inhibitor Cocktail (1861279, ThermoFisher) for snap frozen tissues. Tissues were homogenized at 5500 rpm for 20 s using a Precellys® 24 homogenizer (Bertin Instruments). After disruption, protein lysates were incubated for 30 min at 4 °C. Lysates were centrifuged for 20 min at 15,294*g* to pellet cell debris and supernatant was collected. Protein concentration was measured using Pierce™ BCA Protein Assay Kit (23225, ThermoFisher) according to the manufacturer’s instructions.

### Total RNA extraction and reverse transcription (RT)-PCR

For total RNA isolation, 650 µl of prepared tissue lysate was transferred along with 325 μl Phenol–chloroform–isoamyl alcohol mixture (77617, Sigma-Aldrich) into 5PRIME Phase Lock Gel Heavy tubes (2302830, Quantabio), followed by vigorous shaking for 15 s and centrifugation at 16,000*g* for 5 min. Next, 325 µl of Chloroform–isoamyl alcohol mixture (25666, Sigma-Aldrich) was added and the tubes were shaken again for 15 s, followed by 3 min incubation and centrifugation at 16,000*g* for 5 min. 350 µl of aqueous phase was collected and used to extract total RNA and using AllPrep DNA/RNA 96 Kit (80311, Qiagen). Isolation was performed according to the manufacturer’s instructions with slight modification to remove DNA contamination from the RNA fraction. For this, the RNA fraction was loaded in the RNeasy® 96 Plate and washed with 400 µl RW1 buffer. 80 µl of DNase I (79254, Qiagen) was added to each well and the RNeasy® 96 Plate was incubated for 15 min at room temperature followed by standard protocol starting with washing the 96-well plate with RW1 buffer. Either 500 ng (*dCas9-VPR*, *Ldlr* and *Pcsk9*) or 1 µg (*Serpina1(a-e)*) of total RNA was reverse-transcribed into copy DNA (cDNA) using High-Capacity cDNA Reverse Transcription Kit (4368813, Applied Biosystems).

### Quantitative real-time PCR

Quantitative real-time PCR was performed with a QuantiFast Probe PCR Kit (204256, Qiagen) (for *Serpina1(a-e)*) or TaqMan™ Gene Expression Master Mix (4370074, Applied Biosystems) (for *Ldlr*, *Pcsk9* and *dCas9-VPR* expression) using the following TaqMan™ Gene Expression Assays (Applied Biosystems): Mm01177349_m1 for *Ldlr*, Mm02748447_g1 for *Serpina1a*, Mm04207706_gH for *Serpina1b* and *Serpina1d*, Mm00833655_m1 for *Serpina1e*, Mm00842094_mH for *Serpina1d* and Mm04207703_mH for *Serpina1a*, *Serpina1b*, *Serpina1c*, Mm01263610_m1 for *Pcsk9*, Mm00839502_m1 for *Polr2A*. *dCas9-VPR* expression was analyzed using a Custom TaqMan™ Gene Expression Assay (Applied Biosystems), containing dCas9-Fwd, dCas9-Rev and dCas9-probe (Supplementary Table S1). Relative *Serpina1(a-e)*, *Pcsk9* and *Ldlr* expressions were calculated using 2^−ΔΔCt^ method in relation to *Polr2a*.

### LSL cassette excision PCR

LSL cassette recombination PCR was performed on liver cDNA using Quick-Load® *Taq* Master Mix (M0271S, NEB) with primers p1, p3, p2 (Supplementary Table S1). The PCR products with 492 bp for floxed *LSL-dCas9-VPR* and 393 bp for recombined *dCas9-VPR* products were separated on a 2% E-Gel™ EX Agarose-Gel (G401002, ThermoFisher).

### Genotyping PCR

For genotyping, the *Rosa26* locus was amplified with the PCR primers GenFw1, GenFw2 and GenRev1 (Supplementary Table S1) by using the Taq polymerase High Fidelity (11304011, ThermoFisher). The expected PCR products were 299 bp for wild-type allele and 744 bp long for knock-in allele.

### Quantification of protein expression using Wes™ analysis

Tissue lysates were analyzed using automated Simple Wes system (Protein Simple) with 12–230 kDa Wes Separation Module capillary cartridges (SM-W004, Protein Simple). Anti- mouse (DM-002, Protein Simple), anti-rabbit (DM-001, Protein Simple), anti-goat (DM-006, Protein Simple) detection modules or F(ab’)2 anti-Rat IgG (H + L)-HRPO (1:20, 112-036-062, Jackson Immuno Research) were used, depending on host species of the primary antibodies. The following primary antibodies were used: LDLR (1:50, PAB8804, Abnova), SERPINA1A (1:20, MAB7690, R&D Systems), PCSK9 (1:10, AF3985, R&D Systems) and β-actin (1:20, NB600-501, Novus Biologicals). Specificity of PCSK9 detection was confirmed using recombinant mouse PCSK9 protein (9258-SE-022, R&D Systems). Compass software version 6.0.0 (Protein Simple) was used to analyze the data. Area under the peak of the protein of interest was measured and normalized with respect to the β-actin area under the peak.

### PCSK9 and AAT ELISA

Proteins were determined in plasma using Mouse Proprotein Convertase 9/PCSK9 Quantikine ELISA Kit (MPC900, R&D Systems) and Mouse A1AT ELISA Kit (E-90A1T, Immunology Consultants Laboratory) according to the manufacturer’s instructions detecting all 5 paralogues of *Serpina1*.

### Plasma LDL, HDL, total cholesterol analysis

HDL (high-density lipoprotein), LDL and total cholesterol plasma levels were determined using a COBAS INTEGRA® 400 Plus chemistry analyzer (Roche Diagnostics, Germany), according to the manufacturer's instructions.

### Immunohistochemistry

Mouse tissues were dissected and immediately transferred to 10% neutral buffered formalin (HT501128, Sigma-Aldrich). Tissues were fixed for at least 24 h before samples were processed with an automated tissue processor (Tissue-Tek® VIP® 6, Sakura), embedded in paraffin and cut into 3 µm sections. Immunohistochemical (IHC) staining for LDLR was carried out on the automated Leica Bond RX™ platform (Leica Biosystems, Melbourne, Australia) using a monoclonal rabbit anti-LDL receptor antibody (1:1200, clone EP1553Y, ab271846, abcam) after heat-induced epitope retrieval with Bond™ Epitope Retrieval Solution 1 (ER1, pH6, Leica Biosystems, Newcastle, United Kingdom). Antibody dilution was titrated to have a moderate staining signal in livers of AAV8-Cre-only treated mice. Bound antibodies were visualized using the Bond™ Polymer Refine Detection System (Leica Biosystems, Newcastle, United Kingdom). Anti-LDLR stained sections were scanned with the Axio Scan.Z1 (20 × objective, Carl Zeiss Microscopy GmbH, Jena, Germany).

### Statistics

Statistical analyses were performed using GraphPad Prism 9 (GraphPad). Significance was determined according to the p values as *p < 0.05, **p < 0.01, ***p < 0.001 and ****p < 0.0001. Results are shown as mean values ± s.d. Comparison between experimental groups was made using a nonparametric Mann–Whitney test.

## Supplementary Information

Supplementary Information.

## Data Availability

All data generated or analysed during this study are included in this published article. Detailed nucleotide sequences of all primers, gRNAs and plasmids used in this study can be found in the supplementary data and additionally deposited in the GenBank database (https://www.ncbi.nlm.nih.gov/genbank) via accession numbers (OP099837, OP099838, OP099839, OP099840, OP099841, OP099842, OP099843, OP099844).
