# Supplementary material for: Rosa26-LSL-dCas9-VPR: a versatile mouse model for tissue specific and simultaneous activation of multiple genes for drug discovery
Source: Sci Rep. 2022 Nov 10;12:19268. doi: 10.1038/s41598-022-23127-7 (PMC9649745; doi:10.1038/s41598-022-23127-7)
Supplement: Supplementary file 1 — Supplementary Information. [file 41598_2022_23127_MOESM1_ESM.pdf]

Supplementary Table S1. Primer and gRNA sequences

Name	Sequence (5’-3’)
gLdlr-1	GGGAAGCGGTGAAATTCTGT
gLdlr-2	GGGCGACCCAGGGTGTGGAA
gLdlr-3	GCGGGGGAGGAGTCTGCAAT
gLdlr-4	GCGGCTAAGTGTCCGGGAAG
gLdlr-5	GGCAAGAAGTGTAGGGGTCG
gLdlr-6	GAAAAGAGACGATGTCACAT
gSerpina1a-1	GGCACAGTGGCCAGGGGGAC
gSerpina1a-2	GGCCAGGGGGACAGGCTAGG
gSerpina1a-3	GGCCCCCTAGCCTGTCCCCC
gSerpina1a-4	GCTGACAGGCTGGTGCACAG
gSerpina1a-5	GGGCTGGTGCACAGTGGCCA
gSerpina1a-6	GCTGACAGGAAATTCCTGAC
gSerpina1b-1	GGGCTGGTGCACAGTGGCCA
gSerpina1b-2	GCTGACAGGCTGGTGCACAG
gSerpina1b-3	GCTGACAGGAAATTCCTGAC
gSerpina1b-4	GAGAATGGACTCTGACTGAC
gSerpina1b-5	GCAGGAAATTCCTGACAGGC
gSerpina1b-6	GAGGGGCTAAGTCCATCGAG
gSerpina1c-1	GAGGGGCTAAGTTCATCGAT
gSerpina1c-2	GCAGGTGTCCAAGGTGCGTC
gSerpina1c-3	GCACTCTCCTGGGCAATTCT
gSerpina1c-4	GGTGTCTCCATTCATCAAGT
gSerpina1c-5	GTGCACAGTGGCCAGGGGAC
gSerpina1c-6	GGGCTGGTGCACAGTGGCCA
gSerpina1d-1	GGGGAGAGAGTCCATCCATT
gSerpina1d-2	GGGACATCGGATGTTGTGTT
gSerpina1d-3	GTGTTCGGTTGTGTTTATGA
gSerpina1d-4	GTGCACAGTGGCCAGGGGAC
gSerpina1d-5	GGGCTGGTGCACAGTGGCCA
gSerpina1d-6	GCTGACAGGCTGGTGCACAG
gSerpina1e-1	GTGGGGCTGAAGCATCAAGA
gSerpina1e-2	GCCACTGTTGCTCTTAGAGA
gSerpina1e-3	GACCCCTGCCCCAACCTCGG
gSerpina1e-4	GACGCAGTATGACCCAGCAT
gSerpina1e-5	GCTAAGAGCAACAGTGGCCC
gSerpina1e-6	GAGCCCCCGAGGTTGGGGCA
gPcsk9-1	GAGGCGGGGTGCCAACTCAG
gPcsk9-2	GGGAGTGGGGATTAAGAGGG
gPcsk9-3	GTCAAATGACGCTCAGAGTG
gPcsk9-4	GACGCCACCCCGAGCCCCAT

gPcsk9-5	GTAAGAGGGGGGAATGTAAC
gPcsk9-6	GATTGGAGTGGGGATTAAGA
U6-Fwd	GGACTATCATATGCTTACCGTAACTTGAA
U6-Rev	GTGTTTCGTCCTTTCCACAAGAT
U6-probe	ATAAAGCCAAGAAATCG
LP1-Fwd	GTAGGCGGGCGACTCAGA
LP1-Rev	CCCAGTTATCGGAGGAGCAA
LP1-probe	CCCAGCCAGTGGACTTAGCCCCTG
dCas9-Fwd	ACAAACTGATTCGAGAGGTGAAAGT
dCas9-Rev	GTGGTAATTGTTGATCTCTCTCACCTT
dCas9-probe	CTGGTCTCAGATTTCA
p1	TGGCAGGCTTGAGATCTGG
P2	CCCAAGGCACACAAAAAACC
P3	CGCCGTGCTGTTCTTTTG
GenFw1	TTGGGTCCACTCAGTAGATGC
GenFw2	CTCTTCCCTCGTGATCTGCAACTCC
GenRev1	CATGTCTTTAATCTACCTCGATGG

Supplementary Table S2. Plasmid sequences

AAV:ITR-LP1-Cre-SV40 pA-ITR (AAV8-*Cre*)	ITR, LP1 promoter, NLS, Cre, SV40 polyA
CCTGCAGGCAGCTGCGCGCTCGCTCGCTCACTGAGGCCGCCCGGGCGTCGGGCGACCTTTGGTCGCCCG<br>GCCTCAGTGAGCGAGCGAGCGCGCAGAGAGGGAGTGGCCAACTCCATCACTAGGGGTTCCTACGCGTGC<br>GGCCGCATGCATGTCGACTCGACCCCCTAAAATGGGCAAACATTGCAAGCAAACAGCAAACACACAGCC<br>CTCCCTGCCTGCTGACCTTGGAGCTGGGGCAGAGGTCAGAGACCTCTCTGGGCCCATGCCACCTCCAAC<br>ATCCACTCGACCCCTTGGAATTTCGGTGGAGAGGAGCAGAGGTTGTCCTGGCGTGGTTTAGGTAGTGTG<br>AGAGGGGAATGACTCCTTTCGGTAAGTGCAGTGGAAGCTGTACACTGCCCAGGCAAAGCGTCCGGGCAG<br>CGTAGGCGGGCGACTCAGATCCCAGCCAGTGGACTTAGCCCCTGTTTGCTCCTCCGATAACTGGGGTGA<br>CCTTGGTTAATATTCACCAGCAGCCTCCCCCGTTGCCCCTCTGGATCCACTGCTTAAATACGGACGAGG<br>ACAGGGCCCTGTCTCCTCAGCTTCAGGCACCACCACTGACCTGGGACAGTGAATCCGGACTCTAAGAGG<br>TACCTTCGAAGCTAGCTTAATTAAGAATTCGCCACCATGCCCAAGAAAAAGCGGAAGGTGTCCAACCTG<br>CTGACCGTGCACCAGAACCTGCCCGCCCTGCCTGTGGACGCCACAAGCGACGAAGTGCGGAAGAACCTG<br>ATGGACATGTTCAGAGACAGACAGGCCTTCAGCGAGCACACCTGGAAGATGCTGCTGAGCGTGTGCAGA<br>TCCTGGGCCGCCTGGTGCAAGCTGAACAACAGAAAGTGGTTCCCCGCCGAGCCCGAGGACGTGCGGGAC<br>TACCTGCTGTATCTGCAGGCCAGAGGCCTGGCCGTGAAAACCATCCAGCAGCACCTGGGCCAGCTGAAC<br>ATGCTGCACAGAAGAAGCGGCCTGCCCAGACCCAGCGACAGCAACGCCGTGTCCCTGGTGATGAGAAGG<br>ATCAGAAAAGAAAACGTGGACGCCGGCGAGAGAGCCAAGCAGGCCCTGGCCTTCGAGAGAACCGACTTC<br>GACCAGGTGCGCAGCCTGATGGAAAACAGCGACAGATGCCAGGATATCAGAAACCTGGCCTTCCTGGGA<br>ATCGCCTACAACACCCTGCTGAGAATCGCCGAGATCGCCAGAATCAGAGTGAAGGACATCAGCAGAACC<br>GACGGCGGCAGAATGCTGATCCACATCGGCAGGACCAAGACCCTGGTGTCCACAGCCGGAGTGGAAAAG<br>GCCCTGAGCCTGGGCGTGACCAAGCTGGTGGAAAGATGGATCAGCGTGTCCGGCGTGGCCGACGACCCC<br>AACAACTACCTGTTCTGCAGAGTGCGCAAGAACGGCGTGGCAGCCCCTAGCGCCACCAGCCAGCTGAGC<br>ACAAGAGCCCTGGAAGGCATCTTCGAGGCCACCCACAGACTGATCTACGGCGCCAAGGACGACAGCGGC<br>CAGAGATACCTGGCTTGGAGCGGCCACAGCGCCAGAGTGGGCGCTGCCAGAGATATGGCCAGAGCCGGC<br>GTGTCCATCCCCGAGATCATGCAGGCTGGCGGCTGGACCAACGTGAACATCGTGATGAACTACATCCGG<br>AACCTGGACAGCGAGACAGGCGCCATGGTGCGCCTGCTGGAAGATGGCGACTGAGGATCCAAGCTTCTC<br>GAGGGCGCGCCGGATCATAATCAGCCATACCACATTTGTAGAGGTTTTACTTGCTTTAAAAAACCTCCC<br>ACACCTCCCCCTGAACCTGAAACATAAAATGAATGCAATTGTTGTTGTTAACTTGTTTATTGCAGCTTA<br>TAATGGTTACAAATAAAGCAATAGCATCACAAATTTCACAAATAAAGCATTTTTTTCACTGCATTCTAG<br>TTGTGGTTTGTCCAAACTCATCAATGTATCTTATCATGTCTGGATAGATCTCTCCCTCTCTGCGCGCTC<br>GCTCGCTCACTGAGGCCGGGCGACCAAAGGTCGCCCGACGCCCGGGCTTTGCCCGGGCGGCCTCAGTGA<br>GCGAGCGAGCGCGCAGCTGCCTGCAGGGGC	
AAV:ITR-U6-gLdlr-1-U6-gLdlr-2-U6-gLdlr-3-U6-gLdlr-4-U6-gLdlr-5-U6-gLdlr-6-ITR (AAV8-*gLdlr*)	ITR, U6 promoter, gRNA
CCTGCAGGCAGCTGCGCGCTCGCTCGCTCACTGAGGCCGCCCGGGCGTCGGGCGACCTTTGGTCGCCCG<br>GCCTCAGTGAGCGAGCGAGCGCGCAGAGAGGGAGTGGCCAACTCCATCACTAGGGGTTCCTACGCGTGA<br>GGGCCTATTTCCCATGATTCCTTCATATTTGCATATACGATACAAGGCTGTTAGAGAGATAATTAGAAT<br>TAATTTGACTGTAAACACAAAGATATTAGTACAAAATACGTGACGTAGAAAGTAATAATTTCTTGGGTA<br>GTTTGCAGTTTTAAAATTATGTTTTAAAATGGACTATCATATGCTTACCGTAACTTGAAAGTATTTCGA<br>TTTCTTGGCTTTATATATCTTGTGGAAAGGACGAAACACCGGGAAGCGGTGAAATTCTGTGTTTCAGAG<br>CTATGCTGGAAACAGCATAGCAAGTTGAAATAAGGCTAGTCCGTTATCAACTTGAAAAAGTGGCACCGA<br>GTCGGTGCTTTTTTTGAGGGCCTATTTCCCATGATTCCTTCATATTTGCATATACGATACAAGGCTGTT<br>AGAGAGATAATTAGAATTAATTTGACTGTAAACACAAAGATATTAGTACAAAATACGTGACGTAGAAAG<br>TAATAATTTCTTGGGTAGTTTGCAGTTTTAAAATTATGTTTTAAAATGGACTATCATATGCTTACCGTA<br>ACTTGAAAGTATTTCGATTTCTTGGCTTTATATATCTTGTGGAAAGGACGAAACACCGGGCGACCCAGG<br>GTGTGGAAGTTTCAGAGCTATGCTGGAAACAGCATAGCAAGTTGAAATAAGGCTAGTCCGTTATCAACT<br>TGAAAAAGTGGCACCGAGTCGGTGCTTTTTTTGAGGGCCTATTTCCCATGATTCCTTCATATTTGCATA<br>TACGATACAAGGCTGTTAGAGAGATAATTAGAATTAATTTGACTGTAAACACAAAGATATTAGTACAAA<br>ATACGTGACGTAGAAAGTAATAATTTCTTGGGTAGTTTGCAGTTTTAAAATTATGTTTTAAAATGGACT	

<table>
<tr><td colspan="2">ATCATATGCTTACCGTAACTTGAAAGTATTTCGATTTCTTGGCTTTATATATCTTGTGGAAAGGACGAA
ACACCGCGGGGGAGGAGTCTGCAATGTTTCAGAGCTATGCTGGAAACAGCATAGCAAGTTGAAATAAGG
CTAGTCCGTTATCAACTTGAAAAAGTGGCACCGAGTCGGTGCTTTTTTTGAGGGCCTATTTCCCATGAT
TCCTTCATATTTGCATATACGATACAAGGCTGTTAGAGAGATAATTAGAATTAATTTGACTGTAAACAC
AAAGATATTAGTACAAAATACGTGACGTAGAAAGTAATAATTTCTTGGGTAGTTTGCAGTTTTAAAATT
ATGTTTTAAAATGGACTATCATATGCTTACCGTAACTTGAAAGTATTTCGATTTCTTGGCTTTATATAT
CTTGTGGAAAGGACGAAACACCGCGGCTAAGTGTCCGGGAAGGTTTCAGAGCTATGCTGGAAACAGCAT
AGCAAGTTGAAATAAGGCTAGTCCGTTATCAACTTGAAAAAGTGGCACCGAGTCGGTGCTTTTTTTGAG
GGCCTATTTCCCATGATTCCTTCATATTTGCATATACGATACAAGGCTGTTAGAGAGATAATTAGAATT
AATTTGACTGTAAACACAAAGATATTAGTACAAAATACGTGACGTAGAAAGTAATAATTTCTTGGGTAG
TTTGCAGTTTTAAAATTATGTTTTAAAATGGACTATCATATGCTTACCGTAACTTGAAAGTATTTCGAT
TTCTTGGCTTTATATATCTTGTGGAAAGGACGAAACACCGGCAAGAAGTGTAGGGGTCGGTTTCAGAGC
TATGCTGGAAACAGCATAGCAAGTTGAAATAAGGCTAGTCCGTTATCAACTTGAAAAAGTGGCACCGAG
TCGGTGCTTTTTTTGAGGGCCTATTTCCCATGATTCCTTCATATTTGCATATACGATACAAGGCTGTTA
GAGAGATAATTAGAATTAATTTGACTGTAAACACAAAGATATTAGTACAAAATACGTGACGTAGAAAGT
AATAATTTCTTGGGTAGTTTGCAGTTTTAAAATTATGTTTTAAAATGGACTATCATATGCTTACCGTAA
CTTGAAAGTATTTCGATTTCTTGGCTTTATATATCTTGTGGAAAGGACGAAACACCGAAAAGAGACGAT
GTCACATGTTTCAGAGCTATGCTGGAAACAGCATAGCAAGTTGAAATAAGGCTAGTCCGTTATCAACTT
GAAAAAGTGGCACCGAGTCGGTGCTTTTTTTAGATCTCTCCCTCTCTGCGCGCTCGCTCGCTCACTGAG
GCCGGGCGACCAAAGGTCGCCCGACGCCCGGGCTTTGCCCGGGCGGCCTCAGTGAGCGAGCGAGCGCGC
AGCTGCCTGCAGGGGC</td></tr>
<tr><td>AAV:ITR-U6-gPcsk9-1-U6-gPcsk9-2-U6-gPcsk9-3-U6-gPcsk9-4-U6-gPcsk9-5-U6-gPcsk9-6-ITR (AAV8-gPcsk9)</td><td>ITR, U6 promoter, gRNA</td></tr>
<tr><td colspan="2">CCTGCAGGCAGCTGCGCGCTCGCTCGCTCACTGAGGCCGCCCGGGCGTCGGGCGACCTTTGGTCGCCCG
GCCTCAGTGAGCGAGCGAGCGCGCAGAGAGGGAGTGGCCAACTCCATCACTAGGGGTTCCTACGCGTGA
GGGCCTATTTCCCATGATTCCTTCATATTTGCATATACGATACAAGGCTGTTAGAGAGATAATTAGAAT
TAATTTGACTGTAAACACAAAGATATTAGTACAAAATACGTGACGTAGAAAGTAATAATTTCTTGGGTA
GTTTGCAGTTTTAAAATTATGTTTTAAAATGGACTATCATATGCTTACCGTAACTTGAAAGTATTTCGA
TTTCTTGGCTTTATATATCTTGTGGAAAGGACGAAACACCGAGGCGGGGTGCCAACTCAGGTTTCAGAG
CTATGCTGGAAACAGCATAGCAAGTTGAAATAAGGCTAGTCCGTTATCAACTTGAAAAAGTGGCACCGA
GTCGGTGCTTTTTTTGAGGGCCTATTTCCCATGATTCCTTCATATTTGCATATACGATACAAGGCTGTT
AGAGAGATAATTAGAATTAATTTGACTGTAAACACAAAGATATTAGTACAAAATACGTGACGTAGAAAG
TAATAATTTCTTGGGTAGTTTGCAGTTTTAAAATTATGTTTTAAAATGGACTATCATATGCTTACCGTA
ACTTGAAAGTATTTCGATTTCTTGGCTTTATATATCTTGTGGAAAGGACGAAACACCGGGAGTGGGGAT
TAAGAGGGGTTTCAGAGCTATGCTGGAAACAGCATAGCAAGTTGAAATAAGGCTAGTCCGTTATCAACT
TGAAAAAGTGGCACCGAGTCGGTGCTTTTTTTGAGGGCCTATTTCCCATGATTCCTTCATATTTGCATA
TACGATACAAGGCTGTTAGAGAGATAATTAGAATTAATTTGACTGTAAACACAAAGATATTAGTACAAA
ATACGTGACGTAGAAAGTAATAATTTCTTGGGTAGTTTGCAGTTTTAAAATTATGTTTTAAAATGGACT
ATCATATGCTTACCGTAACTTGAAAGTATTTCGATTTCTTGGCTTTATATATCTTGTGGAAAGGACGAA
ACACCGTCAAATGACGCTCAGAGTGGTTTCAGAGCTATGCTGGAAACAGCATAGCAAGTTGAAATAAGG
CTAGTCCGTTATCAACTTGAAAAAGTGGCACCGAGTCGGTGCTTTTTTTGAGGGCCTATTTCCCATGAT
TCCTTCATATTTGCATATACGATACAAGGCTGTTAGAGAGATAATTAGAATTAATTTGACTGTAAACAC
AAAGATATTAGTACAAAATACGTGACGTAGAAAGTAATAATTTCTTGGGTAGTTTGCAGTTTTAAAATT
ATGTTTTAAAATGGACTATCATATGCTTACCGTAACTTGAAAGTATTTCGATTTCTTGGCTTTATATAT
CTTGTGGAAAGGACGAAACACCGACGCCACCCCGAGCCCCATGTTTCAGAGCTATGCTGGAAACAGCAT
AGCAAGTTGAAATAAGGCTAGTCCGTTATCAACTTGAAAAAGTGGCACCGAGTCGGTGCTTTTTTTGAG
GGCCTATTTCCCATGATTCCTTCATATTTGCATATACGATACAAGGCTGTTAGAGAGATAATTAGAATT
AATTTGACTGTAAACACAAAGATATTAGTACAAAATACGTGACGTAGAAAGTAATAATTTCTTGGGTAG
TTTGCAGTTTTAAAATTATGTTTTAAAATGGACTATCATATGCTTACCGTAACTTGAAAGTATTTCGAT
TTCTTGGCTTTATATATCTTGTGGAAAGGACGAAACACCGTAAGAGGGGGGAATGTAACGTTTCAGAGC</td></tr>
</table>

<table>
<tr><td colspan="2">TATGCTGGAAACAGCATAGCAAGTTGAAATAAGGCTAGTCCGTTATCAACTTGAAAAAGTGGCACCGAG
TCGGTGCTTTTTTTGAGGGCCTATTTCCCATGATTCCTTCATATTTGCATATACGATACAAGGCTGTTA
GAGAGATAATTAGAATTAATTTGACTGTAAACACAAAGATATTAGTACAAAATACGTGACGTAGAAAGT
AATAATTTCTTGGGTAGTTTGCAGTTTTAAAATTATGTTTTAAAATGGACTATCATATGCTTACCGTAA
CTTGAAAGTATTTCGATTTCTTGGCTTTATATATCTTGTGGAAAGGACGAAACACCGATTGGAGTGGG
ATTAAGAGTTTCAGAGCTATGCTGGAAACAGCATAGCAAGTTGAAATAAGGCTAGTCCGTTATCAACTT
GAAAAAGTGGCACCGAGTCGGTGCTTTTTTTAGATCTCTCCCTCTCTGCGCGCTCGCTCGCTCACTGAG
GCCGGGCGACCAAAGGTCGCCCGACGCCCGGGCTTTGCCCGGGCGGCCTCAGTGAGCGAGCGAGCGCGC
AGCTGCCTGCAGGGGC</td></tr>
<tr><td>AAV:ITR-U6-gSerpina1a-1-U6-gSerpina1a-2-U6-gSerpina1a-3-U6-gSerpina1a-4-U6-gSerpina1a-5-U6-gSerpina1a-6-ITR (AAV8-gSerpina1a)</td><td>ITR, U6 promoter, gRNA</td></tr>
<tr><td colspan="2">CCTGCAGGCAGCTGCGCGCTCGCTCGCTCACTGAGGCCGCCCGGGCGTCGGGCGACCTTTGGTCGCCCG
GCCTCAGTGAGCGAGCGAGCGCGCAGAGAGGGAGTGGCCAACTCCATCACTAGGGGTTCCTACGCGTGA
GGGCCTATTTCCCATGATTCCTTCATATTTGCATATACGATACAAGGCTGTTAGAGAGATAATTAGAAT
TAATTTGACTGTAAACACAAAGATATTAGTACAAAATACGTGACGTAGAAAGTAATAATTTCTTGGGTA
GTTTGCAGTTTTAAAATTATGTTTTAAAATGGACTATCATATGCTTACCGTAACTTGAAAGTATTTCGA
TTTCTTGGCTTTATATATCTTGTGGAAAGGACGAAACACCGGCACAGTGGCCAGGGGGACGTTTCAGAG
CTATGCTGGAAACAGCATAGCAAGTTGAAATAAGGCTAGTCCGTTATCAACTTGAAAAAGTGGCACCGA
GTCGGTGCTTTTTTTGAGGGCCTATTTCCCATGATTCCTTCATATTTGCATATACGATACAAGGCTGTT
AGAGAGATAATTAGAATTAATTTGACTGTAAACACAAAGATATTAGTACAAAATACGTGACGTAGAAAG
TAATAATTTCTTGGGTAGTTTGCAGTTTTAAAATTATGTTTTAAAATGGACTATCATATGCTTACCGTA
ACTTGAAAGTATTTCGATTTCTTGGCTTTATATATCTTGTGGAAAGGACGAAACACCGGCCAGGGGGAC
AGGCTAGGGTTTCAGAGCTATGCTGGAAACAGCATAGCAAGTTGAAATAAGGCTAGTCCGTTATCAACT
TGAAAAAGTGGCACCGAGTCGGTGCTTTTTTTGAGGGCCTATTTCCCATGATTCCTTCATATTTGCATA
TACGATACAAGGCTGTTAGAGAGATAATTAGAATTAATTTGACTGTAAACACAAAGATATTAGTACAAA
ATACGTGACGTAGAAAGTAATAATTTCTTGGGTAGTTTGCAGTTTTAAAATTATGTTTTAAAATGGACT
ATCATATGCTTACCGTAACTTGAAAGTATTTCGATTTCTTGGCTTTATATATCTTGTGGAAAGGACGAA
ACACCGGCCCCCTAGCCTGTCCCCCGTTTCAGAGCTATGCTGGAAACAGCATAGCAAGTTGAAATAAGG
CTAGTCCGTTATCAACTTGAAAAAGTGGCACCGAGTCGGTGCTTTTTTTGAGGGCCTATTTCCCATGAT
TCCTTCATATTTGCATATACGATACAAGGCTGTTAGAGAGATAATTAGAATTAATTTGACTGTAAACAC
AAAGATATTAGTACAAAATACGTGACGTAGAAAGTAATAATTTCTTGGGTAGTTTGCAGTTTTAAAATT
ATGTTTTAAAATGGACTATCATATGCTTACCGTAACTTGAAAGTATTTCGATTTCTTGGCTTTATATAT
CTTGTGGAAAGGACGAAACACCGCTGACAGGCTGGTGCACAGGTTTCAGAGCTATGCTGGAAACAGCAT
AGCAAGTTGAAATAAGGCTAGTCCGTTATCAACTTGAAAAAGTGGCACCGAGTCGGTGCTTTTTTTGAG
GGCCTATTTCCCATGATTCCTTCATATTTGCATATACGATACAAGGCTGTTAGAGAGATAATTAGAATT
AATTTGACTGTAAACACAAAGATATTAGTACAAAATACGTGACGTAGAAAGTAATAATTTCTTGGGTAG
TTTGCAGTTTTAAAATTATGTTTTAAAATGGACTATCATATGCTTACCGTAACTTGAAAGTATTTCGAT
TTCTTGGCTTTATATATCTTGTGGAAAGGACGAAACACCGGGCTGGTGCACAGTGGCCAGTTTCAGAGC
TATGCTGGAAACAGCATAGCAAGTTGAAATAAGGCTAGTCCGTTATCAACTTGAAAAAGTGGCACCGAG
TCGGTGCTTTTTTTGAGGGCCTATTTCCCATGATTCCTTCATATTTGCATATACGATACAAGGCTGTTA
GAGAGATAATTAGAATTAATTTGACTGTAAACACAAAGATATTAGTACAAAATACGTGACGTAGAAAGT
AATAATTTCTTGGGTAGTTTGCAGTTTTAAAATTATGTTTTAAAATGGACTATCATATGCTTACCGTAA
CTTGAAAGTATTTCGATTTCTTGGCTTTATATATCTTGTGGAAAGGACGAAACACCGCTGACAGGAAAT
TCCTGACGTTTCAGAGCTATGCTGGAAACAGCATAGCAAGTTGAAATAAGGCTAGTCCGTTATCAACTT
GAAAAAGTGGCACCGAGTCGGTGCTTTTTTTAGATCTCTCCCTCTCTGCGCGCTCGCTCGCTCACTGAG
GCCGGGCGACCAAAGGTCGCCCGACGCCCGGGCTTTGCCCGGGCGGCCTCAGTGAGCGAGCGAGCGCGC
AGCTGCCTGCAGGGGC</td></tr>
</table>

<table>
<tr><td>AAV:ITR-U6-gSerpina1b-1-U6-gSerpina1b-2-U6-gSerpina1b-3-U6-gSerpina1b-4-U6-gSerpina1b-5-U6-gSerpina1b-6-ITR (AAV8-gSerpina1b)</td><td>ITR, U6 promoter, gRNA</td></tr>
<tr><td colspan="2">CCTGCAGGCAGCTGCGCGCTCGCTCGCTCACTGAGGCCGCCCGGGCGTCGGGCGACCTTTGGTCGCCCG
GCCTCAGTGAGCGAGCGAGCGCGCAGAGAGGGAGTGGCCAACTCCATCACTAGGGGTTCCTACGCGTGA
GGGCCTATTTCCCATGATTCCTTCATATTTGCATATACGATACAAGGCTGTTAGAGAGATAATTAGAAT
TAATTTGACTGTAAACACAAAGATATTAGTACAAAATACGTGACGTAGAAAGTAATAATTTCTTGGGTA
GTTTGCAGTTTTAAAATTATGTTTTAAAATGGACTATCATATGCTTACCGTAACTTGAAAGTATTTCGA
TTTCTTGGCTTTATATATCTTGTGGAAAGGACGAAACACCGGGCTGGTGCACAGTGGCCAGTTTCAGAG
CTATGCTGGAAACAGCATAGCAAGTTGAAATAAGGCTAGTCCGTTATCAACTTGAAAAAGTGGCACCGA
GTCGGTGCTTTTTTTGAGGGCCTATTTCCCATGATTCCTTCATATTTGCATATACGATACAAGGCTGTT
AGAGAGATAATTAGAATTAATTTGACTGTAAACACAAAGATATTAGTACAAAATACGTGACGTAGAAAG
TAATAATTTCTTGGGTAGTTTGCAGTTTTAAAATTATGTTTTAAAATGGACTATCATATGCTTACCGTA
ACTTGAAAGTATTTCGATTTCTTGGCTTTATATATCTTGTGGAAAGGACGAAACACCGCTGACAGGCTG
GTGCACAGGTTTCAGAGCTATGCTGGAAACAGCATAGCAAGTTGAAATAAGGCTAGTCCGTTATCAACT
TGAAAAAGTGGCACCGAGTCGGTGCTTTTTTTGAGGGCCTATTTCCCATGATTCCTTCATATTTGCATA
TACGATACAAGGCTGTTAGAGAGATAATTAGAATTAATTTGACTGTAAACACAAAGATATTAGTACAAA
ATACGTGACGTAGAAAGTAATAATTTCTTGGGTAGTTTGCAGTTTTAAAATTATGTTTTAAAATGGACT
ATCATATGCTTACCGTAACTTGAAAGTATTTCGATTTCTTGGCTTTATATATCTTGTGGAAAGGACGAA
ACACCGCTGACAGGAAATTCCTGACGTTTCAGAGCTATGCTGGAAACAGCATAGCAAGTTGAAATAAGG
CTAGTCCGTTATCAACTTGAAAAAGTGGCACCGAGTCGGTGCTTTTTTTGAGGGCCTATTTCCCATGAT
TCCTTCATATTTGCATATACGATACAAGGCTGTTAGAGAGATAATTAGAATTAATTTGACTGTAAACAC
AAAGATATTAGTACAAAATACGTGACGTAGAAAGTAATAATTTCTTGGGTAGTTTGCAGTTTTAAAATT
ATGTTTTAAAATGGACTATCATATGCTTACCGTAACTTGAAAGTATTTCGATTTCTTGGCTTTATATAT
CTTGTGGAAAGGACGAAACACCGAGAATGGACTCTGACTGACGTTTCAGAGCTATGCTGGAAACAGCAT
AGCAAGTTGAAATAAGGCTAGTCCGTTATCAACTTGAAAAAGTGGCACCGAGTCGGTGCTTTTTTTGAG
GGCCTATTTCCCATGATTCCTTCATATTTGCATATACGATACAAGGCTGTTAGAGAGATAATTAGAATT
AATTTGACTGTAAACACAAAGATATTAGTACAAAATACGTGACGTAGAAAGTAATAATTTCTTGGGTAG
TTTGCAGTTTTAAAATTATGTTTTAAAATGGACTATCATATGCTTACCGTAACTTGAAAGTATTTCGAT
TTCTTGGCTTTATATATCTTGTGGAAAGGACGAAACACCGCAGGAAATTCCTGACAGGCGTTTCAGAGC
TATGCTGGAAACAGCATAGCAAGTTGAAATAAGGCTAGTCCGTTATCAACTTGAAAAAGTGGCACCGAG
TCGGTGCTTTTTTTGAGGGCCTATTTCCCATGATTCCTTCATATTTGCATATACGATACAAGGCTGTTA
GAGAGATAATTAGAATTAATTTGACTGTAAACACAAAGATATTAGTACAAAATACGTGACGTAGAAAGT
AATAATTTCTTGGGTAGTTTGCAGTTTTAAAATTATGTTTTAAAATGGACTATCATATGCTTACCGTAA
CTTGAAAGTATTTCGATTTCTTGGCTTTATATATCTTGTGGAAAGGACGAAACACCGAGGGGCTAAGTC
CATCGAGGTTTCAGAGCTATGCTGGAAACAGCATAGCAAGTTGAAATAAGGCTAGTCCGTTATCAACTT
GAAAAAGTGGCACCGAGTCGGTGCTTTTTTTAGATCTCTCCCTCTCTGCGCGCTCGCTCGCTCACTGAG
GCCGGGCGACCAAAGGTCGCCCGACGCCCGGGCTTTGCCCGGGCGGCCTCAGTGAGCGAGCGAGCGCGC
AGCTGCCTGCAGGGGC</td></tr>
<tr><td>AAV:ITR-U6-gSerpina1c-1-U6-gSerpina1c-2-U6-gSerpina1c-3-U6-gSerpina1c-4-U6-gSerpina1c-5-U6-gSerpina1c-6-ITR (AAV8-gSerpina1c)</td><td>ITR, U6 promoter, gRNA</td></tr>
<tr><td colspan="2">CCTGCAGGCAGCTGCGCGCTCGCTCGCTCACTGAGGCCGCCCGGGCGTCGGGCGACCTTTGGTCGCCCG
GCCTCAGTGAGCGAGCGAGCGCGCAGAGAGGGAGTGGCCAACTCCATCACTAGGGGTTCCTACGCGTGA
GGGCCTATTTCCCATGATTCCTTCATATTTGCATATACGATACAAGGCTGTTAGAGAGATAATTAGAAT
TAATTTGACTGTAAACACAAAGATATTAGTACAAAATACGTGACGTAGAAAGTAATAATTTCTTGGGTA
GTTTGCAGTTTTAAAATTATGTTTTAAAATGGACTATCATATGCTTACCGTAACTTGAAAGTATTTCGA
TTTCTTGGCTTTATATATCTTGTGGAAAGGACGAAACACCGAGGGGCTAAGTTCATCGATGTTTCAGAG
CTATGCTGGAAACAGCATAGCAAGTTGAAATAAGGCTAGTCCGTTATCAACTTGAAAAAGTGGCACCGA
GTCGGTGCTTTTTTTGAGGGCCTATTTCCCATGATTCCTTCATATTTGCATATACGATACAAGGCTGTT</td></tr>
</table>

AGAGAGATAATTAGAATTAATTTGACTGTAAACACAAAGATATTAGTACAAAATACGTGACGTAGAAAG<br>TAATAATTTCTTGGGTAGTTTGCAGTTTTAAAATTATGTTTTAAAATGGACTATCATATGCTTACCGTA<br>ACTTGAAAGTATTTCGATTTCTTGGCTTTATATATCTTGTGGAAAGGACGAAACACCGCAGGTGTCCAA<br>GGTGCGTCGTTTCAGAGCTATGCTGGAAACAGCATAGCAAGTTGAAATAAGGCTAGTCCGTTATCAACT<br>TGAAAAAGTGGCACCGAGTCGGTGCTTTTTTTGAGGGCCTATTTCCCATGATTCCTTCATATTTGCATA<br>TACGATACAAGGCTGTTAGAGAGATAATTAGAATTAATTTGACTGTAAACACAAAGATATTAGTACAAA<br>ATACGTGACGTAGAAAGTAATAATTTCTTGGGTAGTTTGCAGTTTTAAAATTATGTTTTAAAATGGACT<br>ATCATATGCTTACCGTAACTTGAAAGTATTTCGATTTCTTGGCTTTATATATCTTGTGGAAAGGACGAA<br>ACACCGCACTCTCCTGGGCAATTCTGTTTCAGAGCTATGCTGGAAACAGCATAGCAAGTTGAAATAAGG<br>CTAGTCCGTTATCAACTTGAAAAAGTGGCACCGAGTCGGTGCTTTTTTTGAGGGCCTATTTCCCATGAT<br>TCCTTCATATTTGCATATACGATACAAGGCTGTTAGAGAGATAATTAGAATTAATTTGACTGTAAACAC<br>AAAGATATTAGTACAAAATACGTGACGTAGAAAGTAATAATTTCTTGGGTAGTTTGCAGTTTTAAAATT<br>ATGTTTTAAAATGGACTATCATATGCTTACCGTAACTTGAAAGTATTTCGATTTCTTGGCTTTATATAT<br>CTTGTGGAAAGGACGAAACACCGGTGTCTCCATTCATCAAGTGTTTCAGAGCTATGCTGGAAACAGCAT<br>AGCAAGTTGAAATAAGGCTAGTCCGTTATCAACTTGAAAAAGTGGCACCGAGTCGGTGCTTTTTTTGAG<br>GGCCTATTTCCCATGATTCCTTCATATTTGCATATACGATACAAGGCTGTTAGAGAGATAATTAGAATT<br>AATTTGACTGTAAACACAAAGATATTAGTACAAAATACGTGACGTAGAAAGTAATAATTTCTTGGGTAG<br>TTTGCAGTTTTAAAATTATGTTTTAAAATGGACTATCATATGCTTACCGTAACTTGAAAGTATTTCGAT<br>TTCTTGGCTTTATATATCTTGTGGAAAGGACGAAACACCGTGCACAGTGGCCAGGGGACGTTTCAGAGC<br>TATGCTGGAAACAGCATAGCAAGTTGAAATAAGGCTAGTCCGTTATCAACTTGAAAAAGTGGCACCGAG<br>TCGGTGCTTTTTTTGAGGGCCTATTTCCCATGATTCCTTCATATTTGCATATACGATACAAGGCTGTTA<br>GAGAGATAATTAGAATTAATTTGACTGTAAACACAAAGATATTAGTACAAAATACGTGACGTAGAAAGT<br>AATAATTTCTTGGGTAGTTTGCAGTTTTAAAATTATGTTTTAAAATGGACTATCATATGCTTACCGTAA<br>CTTGAAAGTATTTCGATTTCTTGGCTTTATATATCTTGTGGAAAGGACGAAACACCGGGCTGGTGCACA<br>GTGGCCAGTTTCAGAGCTATGCTGGAAACAGCATAGCAAGTTGAAATAAGGCTAGTCCGTTATCAACTT<br>GAAAAAGTGGCACCGAGTCGGTGCTTTTTTTAGATCTCTCCCTCTCTGCGCGCTCGCTCGCTCACTGAG<br>GCCGGGCGACCAAAGGTCGCCCGACGCCCGGGCTTTGCCCGGGCGGCCTCAGTGAGCGAGCGAGCGCGC<br>AGCTGCCTGCAGGGGC	
AAV:ITR-U6-gSerpina1d-1-U6-gSerpina1d-2-U6-gSerpina1d-3-U6-gSerpina1d-4-U6-gSerpina1d-5-U6-gSerpina1d-6-ITR (AAV8-*gSerpina1d*)	ITR, U6 promoter, gRNA
CCTGCAGGCAGCTGCGCGCTCGCTCGCTCACTGAGGCCGCCCGGGCGTCGGGCGACCTTTGGTCGCCCG<br>GCCTCAGTGAGCGAGCGAGCGCGCAGAGAGGGAGTGGCCAACTCCATCACTAGGGGTTCCTACGCGTGA<br>GGGCCTATTTCCCATGATTCCTTCATATTTGCATATACGATACAAGGCTGTTAGAGAGATAATTAGAAT<br>TAATTTGACTGTAAACACAAAGATATTAGTACAAAATACGTGACGTAGAAAGTAATAATTTCTTGGGTA<br>GTTTGCAGTTTTAAAATTATGTTTTAAAATGGACTATCATATGCTTACCGTAACTTGAAAGTATTTCGA<br>TTTCTTGGCTTTATATATCTTGTGGAAAGGACGAAACACCGGGGAGAGAGTCCATCCATTGTTTCAGAG<br>CTATGCTGGAAACAGCATAGCAAGTTGAAATAAGGCTAGTCCGTTATCAACTTGAAAAAGTGGCACCGA<br>GTCGGTGCTTTTTTTGAGGGCCTATTTCCCATGATTCCTTCATATTTGCATATACGATACAAGGCTGTT<br>AGAGAGATAATTAGAATTAATTTGACTGTAAACACAAAGATATTAGTACAAAATACGTGACGTAGAAAG<br>TAATAATTTCTTGGGTAGTTTGCAGTTTTAAAATTATGTTTTAAAATGGACTATCATATGCTTACCGTA<br>ACTTGAAAGTATTTCGATTTCTTGGCTTTATATATCTTGTGGAAAGGACGAAACACCGGGACATCGGAT<br>GTTGTGTTGTTTCAGAGCTATGCTGGAAACAGCATAGCAAGTTGAAATAAGGCTAGTCCGTTATCAACT<br>TGAAAAAGTGGCACCGAGTCGGTGCTTTTTTTGAGGGCCTATTTCCCATGATTCCTTCATATTTGCATA<br>TACGATACAAGGCTGTTAGAGAGATAATTAGAATTAATTTGACTGTAAACACAAAGATATTAGTACAAA<br>ATACGTGACGTAGAAAGTAATAATTTCTTGGGTAGTTTGCAGTTTTAAAATTATGTTTTAAAATGGACT<br>ATCATATGCTTACCGTAACTTGAAAGTATTTCGATTTCTTGGCTTTATATATCTTGTGGAAAGGACGAA<br>ACACCGTGTTCGGTTGTGTTTATGAGTTTCAGAGCTATGCTGGAAACAGCATAGCAAGTTGAAATAAGG<br>CTAGTCCGTTATCAACTTGAAAAAGTGGCACCGAGTCGGTGCTTTTTTTGAGGGCCTATTTCCCATGAT<br>TCCTTCATATTTGCATATACGATACAAGGCTGTTAGAGAGATAATTAGAATTAATTTGACTGTAAACAC<br>AAAGATATTAGTACAAAATACGTGACGTAGAAAGTAATAATTTCTTGGGTAGTTTGCAGTTTTAAAATT	

ATGTTTTAAAATGGACTATCATATGCTTACCGTAACTTGAAAGTATTTCGATTTCTTGGCTTTATATAT<br>CTTGTGGAAAGGACGAAACACCGTGCACAGTGGCCAGGGGACGTTTCAGAGCTATGCTGGAAACAGCAT<br>AGCAAGTTGAAATAAGGCTAGTCCGTTATCAACTTGAAAAAGTGGCACCGAGTCGGTGCTTTTTTTGAG<br>GGCCTATTTCCCATGATTCCTTCATATTTGCATATACGATACAAGGCTGTTAGAGAGATAATTAGAATT<br>AATTTGACTGTAAACACAAAGATATTAGTACAAAATACGTGACGTAGAAAGTAATAATTTCTTGGGTAG<br>TTTGCAGTTTTAAAATTATGTTTTAAAATGGACTATCATATGCTTACCGTAACTTGAAAGTATTTCGAT<br>TTCTTGGCTTTATATATCTTGTGGAAAGGACGAAACACCGGGCTGGTGCACAGTGGCCAGTTTCAGAGC<br>TATGCTGGAAACAGCATAGCAAGTTGAAATAAGGCTAGTCCGTTATCAACTTGAAAAAGTGGCACCGAG<br>TCGGTGCTTTTTTTGAGGGCCTATTTCCCATGATTCCTTCATATTTGCATATACGATACAAGGCTGTTA<br>GAGAGATAATTAGAATTAATTTGACTGTAAACACAAAGATATTAGTACAAAATACGTGACGTAGAAAGT<br>AATAATTTCTTGGGTAGTTTGCAGTTTTAAAATTATGTTTTAAAATGGACTATCATATGCTTACCGTAA<br>CTTGAAAGTATTTCGATTTCTTGGCTTTATATATCTTGTGGAAAGGACGAAACACCGCTGACAGGCTGG<br>TGCACAGGTTTCAGAGCTATGCTGGAAACAGCATAGCAAGTTGAAATAAGGCTAGTCCGTTATCAACTT<br>GAAAAAGTGGCACCGAGTCGGTGCTTTTTTTAGATCTCTCCCTCTCTGCGCGCTCGCTCGCTCACTGAG<br>GCCGGGCGACCAAAGGTCGCCCGACGCCCGGGCTTTGCCCGGGCGGCCTCAGTGAGCGAGCGAGCGCGC<br>AGCTGCCTGCAGGGGC	
AAV:ITR-U6-gSerpina1e-1-U6-gSerpina1e-2-U6-gSerpina1e-3-U6-gSerpina1e-4-U6-gSerpina1e-5-U6-gSerpina1e-6-ITR (AAV8-*gSerpina1e*)	ITR, U6 promoter, gRNA
CCTGCAGGCAGCTGCGCGCTCGCTCGCTCACTGAGGCCGCCCGGGCGTCGGGCGACCTTTGGTCGCCCG<br>GCCTCAGTGAGCGAGCGAGCGCGCAGAGAGGGAGTGGCCAACTCCATCACTAGGGGTTCCTACGCGTGA<br>GGGCCTATTTCCCATGATTCCTTCATATTTGCATATACGATACAAGGCTGTTAGAGAGATAATTAGAAT<br>TAATTTGACTGTAAACACAAAGATATTAGTACAAAATACGTGACGTAGAAAGTAATAATTTCTTGGGTA<br>GTTTGCAGTTTTAAAATTATGTTTTAAAATGGACTATCATATGCTTACCGTAACTTGAAAGTATTTCGA<br>TTTCTTGGCTTTATATATCTTGTGGAAAGGACGAAACACCGTGGGGCTGAAGCATCAAGAGTTTCAGAG<br>CTATGCTGGAAACAGCATAGCAAGTTGAAATAAGGCTAGTCCGTTATCAACTTGAAAAAGTGGCACCGA<br>GTCGGTGCTTTTTTTGAGGGCCTATTTCCCATGATTCCTTCATATTTGCATATACGATACAAGGCTGTT<br>AGAGAGATAATTAGAATTAATTTGACTGTAAACACAAAGATATTAGTACAAAATACGTGACGTAGAAAG<br>TAATAATTTCTTGGGTAGTTTGCAGTTTTAAAATTATGTTTTAAAATGGACTATCATATGCTTACCGTA<br>ACTTGAAAGTATTTCGATTTCTTGGCTTTATATATCTTGTGGAAAGGACGAAACACCGCCACTGTTGCT<br>CTTAGAGAGTTTCAGAGCTATGCTGGAAACAGCATAGCAAGTTGAAATAAGGCTAGTCCGTTATCAACT<br>TGAAAAAGTGGCACCGAGTCGGTGCTTTTTTTGAGGGCCTATTTCCCATGATTCCTTCATATTTGCATA<br>TACGATACAAGGCTGTTAGAGAGATAATTAGAATTAATTTGACTGTAAACACAAAGATATTAGTACAAA<br>ATACGTGACGTAGAAAGTAATAATTTCTTGGGTAGTTTGCAGTTTTAAAATTATGTTTTAAAATGGACT<br>ATCATATGCTTACCGTAACTTGAAAGTATTTCGATTTCTTGGCTTTATATATCTTGTGGAAAGGACGAA<br>ACACCGACCCCTGCCCCAACCTCGGGTTTCAGAGCTATGCTGGAAACAGCATAGCAAGTTGAAATAAGG<br>CTAGTCCGTTATCAACTTGAAAAAGTGGCACCGAGTCGGTGCTTTTTTTGAGGGCCTATTTCCCATGAT<br>TCCTTCATATTTGCATATACGATACAAGGCTGTTAGAGAGATAATTAGAATTAATTTGACTGTAAACAC<br>AAAGATATTAGTACAAAATACGTGACGTAGAAAGTAATAATTTCTTGGGTAGTTTGCAGTTTTAAAATT<br>ATGTTTTAAAATGGACTATCATATGCTTACCGTAACTTGAAAGTATTTCGATTTCTTGGCTTTATATAT<br>CTTGTGGAAAGGACGAAACACCGACGCAGTATGACCCAGCATGTTTCAGAGCTATGCTGGAAACAGCAT<br>AGCAAGTTGAAATAAGGCTAGTCCGTTATCAACTTGAAAAAGTGGCACCGAGTCGGTGCTTTTTTTGAG<br>GGCCTATTTCCCATGATTCCTTCATATTTGCATATACGATACAAGGCTGTTAGAGAGATAATTAGAATT<br>AATTTGACTGTAAACACAAAGATATTAGTACAAAATACGTGACGTAGAAAGTAATAATTTCTTGGGTAG<br>TTTGCAGTTTTAAAATTATGTTTTAAAATGGACTATCATATGCTTACCGTAACTTGAAAGTATTTCGAT<br>TTCTTGGCTTTATATATCTTGTGGAAAGGACGAAACACCGCTAAGAGCAACAGTGGCCCGTTTCAGAGC<br>TATGCTGGAAACAGCATAGCAAGTTGAAATAAGGCTAGTCCGTTATCAACTTGAAAAAGTGGCACCGAG<br>TCGGTGCTTTTTTTGAGGGCCTATTTCCCATGATTCCTTCATATTTGCATATACGATACAAGGCTGTTA<br>GAGAGATAATTAGAATTAATTTGACTGTAAACACAAAGATATTAGTACAAAATACGTGACGTAGAAAGT<br>AATAATTTCTTGGGTAGTTTGCAGTTTTAAAATTATGTTTTAAAATGGACTATCATATGCTTACCGTAA<br>CTTGAAAGTATTTCGATTTCTTGGCTTTATATATCTTGTGGAAAGGACGAAACACCGAGCCCCCGAGGT	

TGGGGCAGTTTCAGAGCTATGCTGGAAACAGCATAGCAAGTTGAAATAAGGCTAGTCCGTTATCAACTT
GAAAAAGTGGCACCGAGTCGGTGCTTTTTTTAGATCTCTCCCTCTCTGCGCGCTCGCTCGCTCACTGAG
GCCGGGCGACCAAAGGTCGCCCGACGCCCGGGCTTTGCCCGGGCGGCCTCAGTGAGCGAGCGAGCGCGC
AGCTGCCTGCAGGGGC

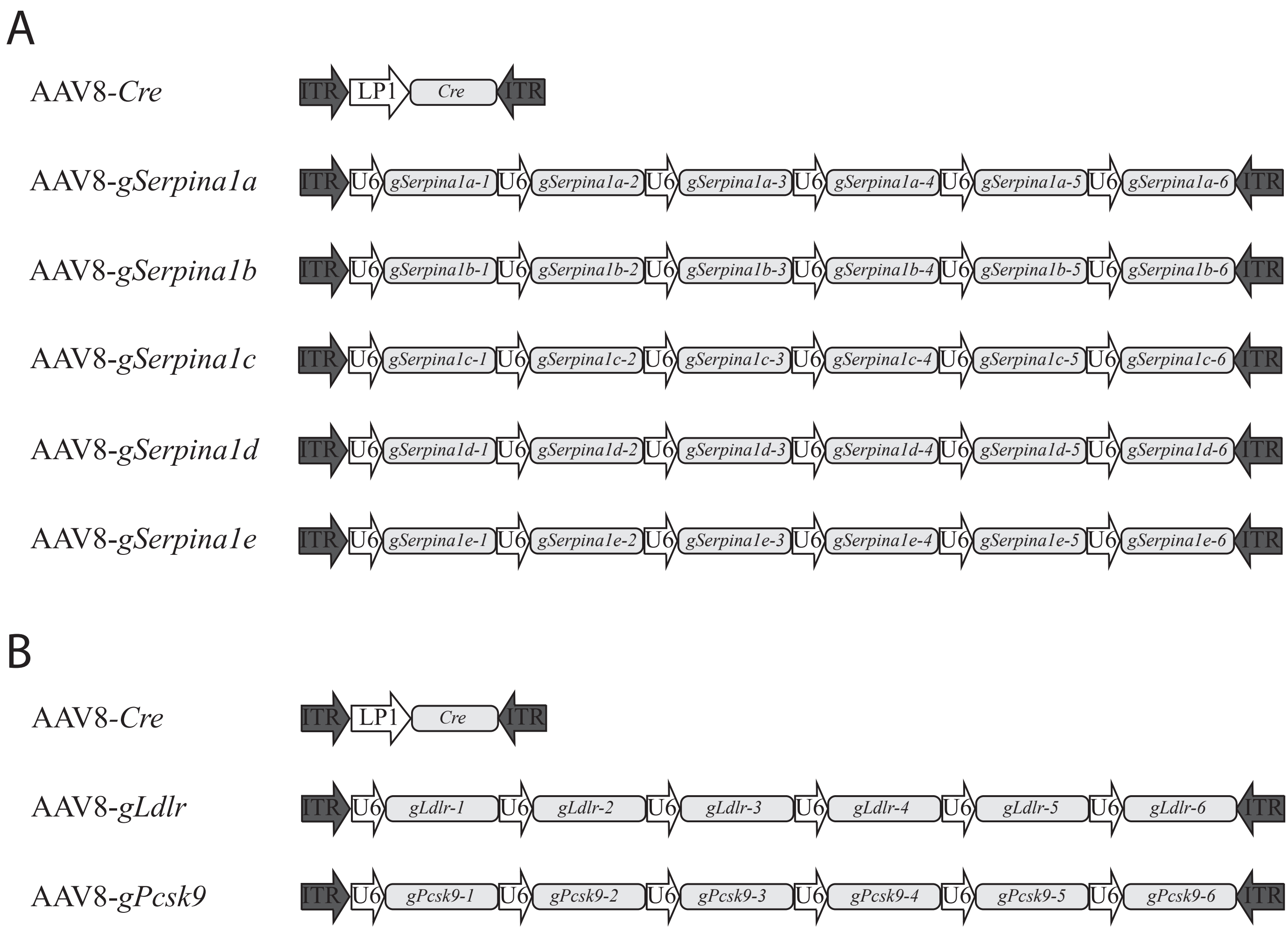

Supplementary Figure 1. Singleplex Serpina1(a-e) and multiplex Ldlr and Pcsk9 overexpression *in vivo* using AAV8-transduced CRISPRa mice.
(A) Rosa26-LSL-dCas9-VPR mice were transduced with AAV8 encoding LP1 promotor driven Cre recombinase (AAV-*Cre*) alone, as a control, or in combination with AAV8 encoding six U6 promoter driven gRNAs against each of the five *Serpina1* gene variants (AAV8-*Serpina1(a-e)*).
(B) Rosa26-LSL-dCas9-VPR mice were iv treated with AAV8 encoded LP1 promoter driven Cre recombinase alone (AAV8-*Cre*) alone, or in combination with AAV8 encoding six U6 promoter driven gRNAs against LDLR (AAV8-*gLdlr*) or PCSK9 (AAV8-*gPcsk9*) or a mixture of both.

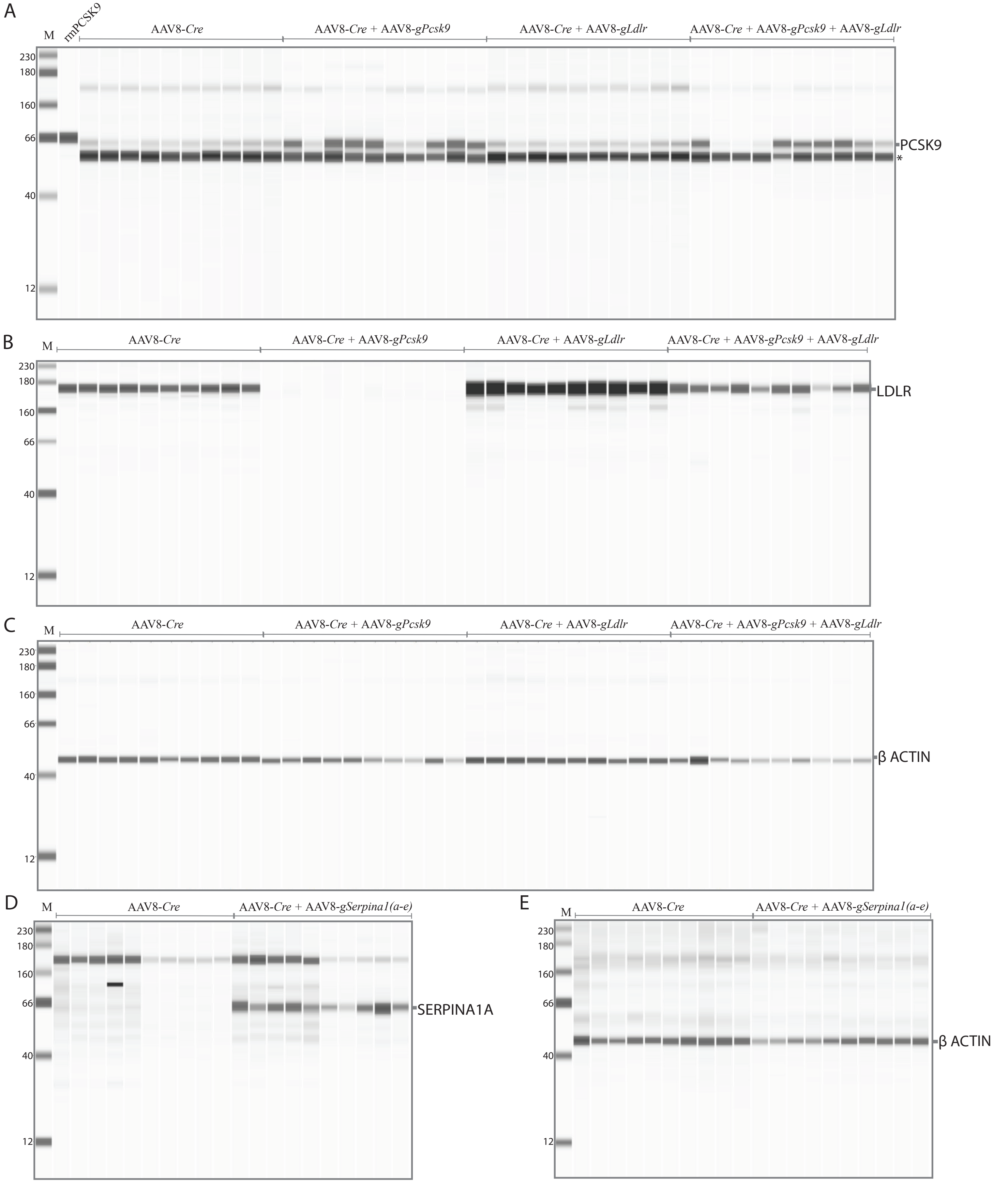

Supplementary Figure S2. Singleplex Serpina1(a-e) and multiplex Ldlr and Pcsk9 overexpression *in vivo* using AAV8-transduced CRISPRa mice.

(A) Pcsk9 protein expression in liver tissue dissected from Rosa26-LSL-dCas9-VPR mice. Animals were iv treated with AAV8-*Cre* alone, or in combination with AAV8-*gLdlr*, AAV8-*gPcsk9* or a mixture of both. For the visualization of the digital data used for the analysis a virtual blot-like image was created. Each lane represents a capillary where the individual reaction was performed. Unspecific bands are indicated with *. rmPCSK9 was used as a postive control.

(B) Ldlr protein expression in liver tissue dissected from Rosa26-LSL-dCas9-VPR mice. Animals were iv treated with AAV8-*Cre* alone, or in combination with AAV8-*gLdlr*, AAV8-*gPcsk9* or a mixture of both. For the visualization of the digital data used for the analysis a virtual blot-like image was created. Each lane represents a capillary where the individual reaction was performed.

(C) β-actin protein expression in liver tissue dissected from Rosa26-LSL-dCas9-VPR mice. Animals were iv treated with AAV8-*Cre* alone, or in combination with AAV8-*gLdlr*, AAV8-*gPcsk9* or a mixture of both. For the visualization of the digital data used for the analysis a virtual blot-like image was created. Each lane represents a capillary where the individual reaction was performed.

(D) Serpina1a protein expression in liver tissue dissected from Rosa26-LSL-dCas9-VPR mice. Animals were iv treated with AAV8-*Cre* alone, or in combination with AAV8-*gSerpina1(a-e)*. For the visualization of the digital data used for the analysis a virtual blot-like image was created. Each lane represents a capillary where the individual reaction was performed.

(E) β-actin protein expression in liver tissue dissected from Rosa26-LSL-dCas9-VPR mice. Animals were iv treated with AAV8-*Cre* alone, or in combination with AAV8-*gSerpina1(a-e)*. For the visualization of the digital data used for the analysis a virtual blot-like image was created. Each lane represents a capillary where the individual reaction was performed.

A

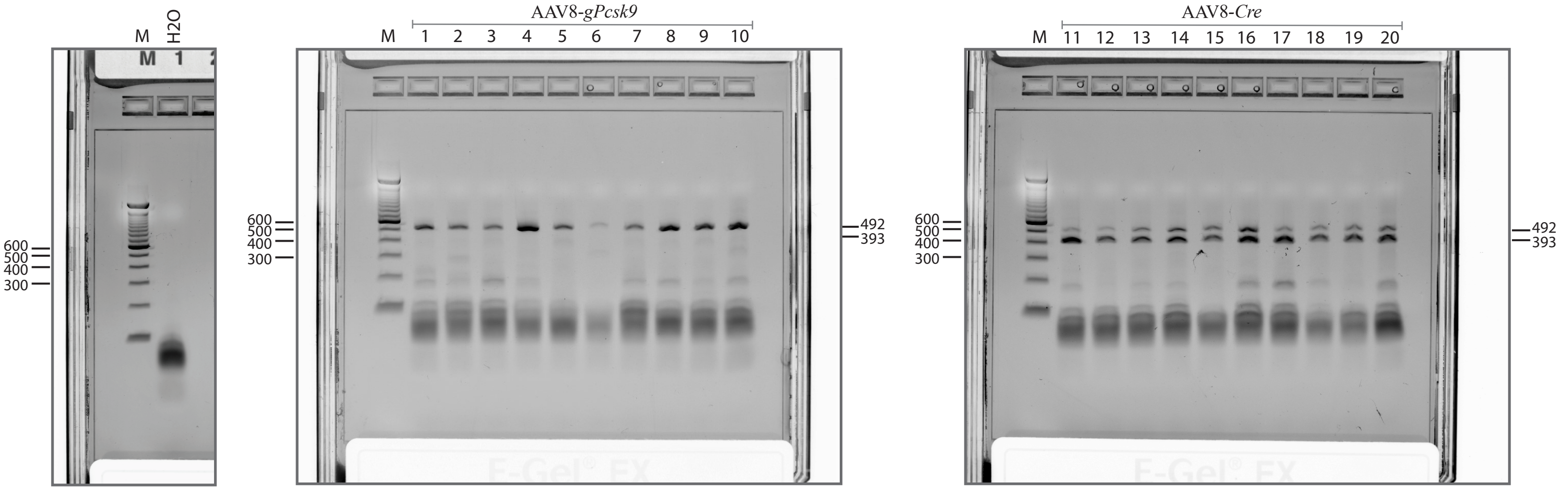

492 bp LSL cassette is not excised
393 bp LSL cassette is excised

B

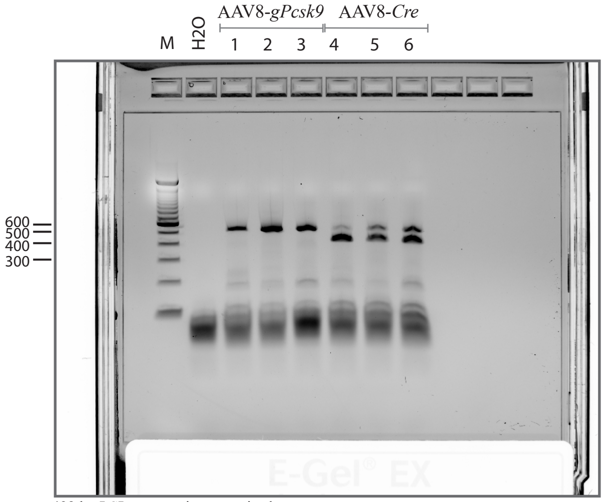

492 bp LSL cassette is not excised
393 bp LSL cassette is excised

Supplementary Figure S3. Generation and Characterization of Cre-Dependent dCas9-VPR-Expressing Mice.

(A) PCR representation showing LSL cassette recombination in liver tissues isolated from Rosa26-LSL-dCas9-VPR mice transduced with AAV8-Cre or AAV8-sgPCSK9 alone. Agarose gel electrophoresis image is shown. Lane 1-10 contains amplicons obtained from tissue samples of different mice treated with AAV8-gPcsk9 and lanes 11-20 from tissue samples of different mice treated with AAV8-Cre. The expected size of PCR products, marker (M) and NTC (H2O) are indicated.

(B) PCR representation showing LSL cassette recombination in liver tissues isolated from Rosa26-LSL-dCas9-VPR mice transduced with AAV8-Cre or AAV8-sgPCSK9 alone. Agarose gel electrophoresis image is shown. Lane 1-3 contains amplicons obtained from tissue samples of different mice treated with AAV8-gPcsk9 and lanes 4-6 from tissue samples of different mice treated with AAV8-Cre. The expected size of PCR products, marker (M) and NTC (H2O) are indicated.
